# Evaluation of the gastrotolerability of ketoprofen, lysine, and gabapentin co-crystal administration in an *in vitro* model of gastric epithelium: a proteomic update

**DOI:** 10.1371/journal.pone.0328496

**Published:** 2025-07-29

**Authors:** Francesco D’Egidio, Laura Brandolini, Vanessa Castelli, Massimiliano Quintiliani, Andrea Aramini, Annamaria Cimini, Marcello Allegretti, Michele d’Angelo

**Affiliations:** 1 Department of Life, Health and Environmental Sciences, University of L’Aquila, L’Aquila, Italy; 2 Dompé Farmaceutici Spa, L’Aquila, Italy; 3 Sbarro Institute for Cancer Research and Molecular Medicine, Temple University, Philadelphia, United States of America; Mass Eye Infirmary, Harvard Medical School / Northeastern University, UNITED STATES OF AMERICA

## Abstract

Chronic pain is a distressful condition that impacts strongly on people’s health, and, to date, no cure has been found. However, several strategies against pain have been proposed. Promising data regarding the usage of nonsteroidal anti-inflammatory drugs (NSAIDs) in combination with gabapentin in pain management laid the foundations for more complex approaches. A recently published study proposed a multimodal approach based on ketoprofen lysine salt (KLS) combined with gabapentin (GABA) in the context of chronic pain. Experiments on *in vitro* models showed supra-additive effects in modulating key pathways involved in neuropathic pain and gastric mucosal damage. Thus, the co-crystallization of ketoprofen, lysine, and gabapentin led to a new ternary drug-drug co-crystal (KLS-GABA co-crystal) to better take advantage of such effects. The new compound showed positive features in *in vitro* and *in vivo* pain models, particularly at the gastrointestinal level. To better understand the gastric impact of the co-crystal we chose to analyze proteomic fluctuations that occur in an *in vitro* model of gastric epithelium upon ethanol injury, aiming to observe the gastric effects of KLS-GABA co-crystal’s administration in comparison with KLS or GABA alone or co-administered as in the multimodal approach. Thus, we performed a 2-dimensional gel electrophoresis (2DE) to compare proteomes from lysates of NCI-N87 cells, chosen as model of gastric epithelium. Among all the localized spots (n = 117), the differentially abundant ones have been filtered and excised (n = 24) to perform mass spectrometry. A total of 414 non-redundant proteins have been found in the excised spots analyzed. A Gene Ontology-based enrichment analysis identified the proteins involved in biological processes, cellular components, and pathways. We then compared the 2DE findings with the western blot analysis confirming the differential proteomic fluctuations in the model. The methodology described here provides a broader picture of the effects of KLS, GABA, and KLS-GABA co-crystal administration in the ethanol-gastric injury model, identifying processes not revealed by other studies by showing proteomic changes and related mechanisms in detail, particularly via modulation of the oxidative stress-related GSTP1 which suggests the higher gastric tolerability of KLS-GABA co-crystal in the analyzed model highlighting its clinical reliability.

## Introduction

Chronic pain is a distressful condition that impacts strongly on people’s physical and psychological health. Chronic pain can be classified as neuropathic, inflammatory, and neuroplastic pain and it is characterized by peripheral and central pathophysiological mechanisms. In particular, the peripheral processes are different among the pain subtypes but, over time, each type of chronic pain can share common central features [1]. Neuropathic pain is characterized by inflammatory and neuropathic features; In fact, neuropathic pain, but also central sensitization, can be also caused by the prolongation of neuroinflammatory states or events of peripheral sensitization eventually induced by inflammatory pain [2]. Chronic pain affects more than 30% of people in the whole world and to date, no cure has been found able to fully counteract its multifactorial pathophysiology. However, researchers managed to find several reliable strategies against pain. Where the monotherapy with a single target failed, the multimodal analgesia to cover multiple targets obtained good results, becoming a popular therapeutic strategy for chronic pain [3–5]. In this context, NSAIDs play a pivotal role. NSAIDs exert anti-inflammatory and analgesic effects, accounting for 8% of prescriptions worldwide. Promising data regarding usage of the NSAID Ketoprofen, usually available as lysine salt (ketoprofen lysine salt, KLS), in combination with gabapentin (GABA), an anti-epileptic agent with strong central and peripheral anti-allodynic activity in neuropathic pain, pave the way for more complex approaches [6–8]. A recent paper proposed a combination of KLS and GABA to obtain an effective multimodal therapeutic approach for chronic pain [9]. Experiments in *in vitro* models showed supra-additive effects at molecular level of the neuroinflammatory cascade and also an improved ketoprofen’s gastrotolerability profile. Thus, a proposed co-crystallization of ketoprofen, lysine and gabapentin led to a new ternary drug-drug co-crystal (KLS-GABA co-crystal) with the aim to exploit their supra-additive effects. The new compound has been widely tested, showing remarkable positive features in several *in vitro* and *in vivo* pain models. Among all the features, the co-crystal is characterized by an improved gastrointestinal tolerability in both inflammatory and chronic neuropathic pain models in rats [9]. However, complications related to the use of these drugs can always occur particularly at the gastric level [10]. Considering this, knowing the reliability of the tested compound and its clinical potential, we chose to deepen the knowledge around the gastric impact of the co-crystal through a proteomic approach in order to better support the knowledge around the proposed drug. We opted for a model of leaky gut already used in our previous study [9]. Specifically, we selected an ethanol-induced injury model using NCI-N87 cells, a well characterized *in vitro* system already used to outline the gastrotolerability of different NSAIDs, among which KLS and GABA [9,11,12]. As ethanol appeared to be a potent gastrolesive agent, its gastric effects have been deeply described both at the morphological and molecular levels, showing the involvement of inflammatory mediators, oxidative stress, lipid peroxidation and, ultimately, cell death [13–16]. The aim of the current study was to analyze the proteomic fluctuations that can occur in the *in vitro* model of leaky gut upon ethanol injury, and subsequent co-crystal KLS-GABA treatment in comparison with KLS and GABA alone or co-administered as in the multimodal approach.

## Materials and methods

### Cell culture and treatment

As a model of leaky gut, human gastric carcinoma NCI-N87 cells (ATCC, USA) were used, passage numbers were between 2–6. Cells were cultured in RPMI 1640 MEDIUM (Sigma, USA) supplemented with 10% heat-inactivated fetal bovine serum and 1% glutamine (Sigma, USA; no antibiotics) at 37 °C in a 5% CO2-humified incubator. The culture medium was refreshed every 2 days until reached 70–80% confluency. Then, cells were harvested using trypsin-EDTA solution, seeded at a density of about 2.5 × 10^5^ cell/cm^2^, and cultured for up to 25 days post-confluency renewing the medium every 2 days. Cells were treated for 24 h with ethanol 6% (Sigma) and 72 h with KLS, GABA, the combination of KLS+GABA, and KLS-GABA co-crystal (KLS-GABA) with ethanol [11]. KLS stock solution (25 mM) was freshly prepared by dissolving the powder in sterile water and then it was used at the final concentration of 800 μМ diluted in cell culture media. GABA stock solution (25 mM) was freshly prepared by dissolving the powder in sterile water and then it was used at the final concentration of 800 μМ diluted in cell culture media. The cocrystal KLS-GABA was freshly prepared by dissolving the crystal in a sterile culture medium at the final concentration of 800 μМ.

### Two-dimensional gel electrophoresis (2DE)

To separate protein samples by pI and MW, the following protocol was followed. Solubilization and extraction of proteins have been achieved on NCI-N87 cells via lysis in urea lysis buffer (7 M urea, 2 M thiourea, 4% w/v CHAPS, and 40 mM DTT, 0.2% w/v ampholytes pH 3–10). The protein concentration was estimated by reducing agent and detergent compatible (RC-DC) protein assay kit (#500–0121, Biorad), and the readings were taken at 750 nm in a microplate reader (Spark, Tecan). To remove contaminants, such as salts, ampholytes, and lipids, the ReadyPrep 2-D Cleanup Kit (#163–2130, Biorad) has been used. With this kit, up to 500 μg of each protein sample has been processed. The precipitated proteins were suspended in 150 µL of ReadyPrep Rehydration/Sample buffer (8 M urea, 2% CHAPS, 50 mM DTT, 0.2% w/v BioLyte 3/10 ampholytes, and bromophenol blue in trace) (#163–2106, Biorad). Then, ReadyStrip IPG strips (7 cm, pH 3–10NL, #163–2002) were rehydrated overnight at 25°C with a rehydration buffer containing protein samples. The proteins on the strips were then focused on an IEF unit Ettan IPGphor (Amersham Biosciences) at 20°C. The IEF program was the following: 1000 V, 1 hour and 30 minutes gradient; 1000 V, 30 minutes step-n-hold; 4000 V, 1 hour and 30 minutes gradient; 4000 V, 2 hours step-n-hold. The program has been done with the maximum current limit of 50 µA/strip throughout the procedure. To pass from the first to the second dimension, reduction and alkylation of the focused proteins on the strips have been performed. Following IEF, the IPG strips were kept in trays (#165–4035, Biorad) and equilibrated in Equilibration buffer I (6 M urea, 2% SDS, 0.375 M Tris-HCL [pH 8.8], 20% glycerol, and 2% w/v DTT) (#163–2107, Biorad) for 12 minutes at 25°C with continuous gentle shaking. The strips were again equilibrated in Equilibration buffer II (2.5% w/v Iodoacetamide in place of DTT in buffer I) (#163–2108) for 5 minutes. After equilibration, strips were washed in distilled water. Proteins were separated in the second dimension on Mini-PROTEAN TGX Precast Gels (4–20% gradient, #456–1091, Biorad) in a vertical electrophoresis unit (Mini-PROTEAN Tetra Cell, #1658004, Bio-Rad) at a constant voltage of 200 V until the dye front reached the bottom of the gel. The gels were fixed in a solution of 40% v/v ethanol and 10% v/v acetic acid for 15 minutes with continuous gentle shaking and then stained with QC Colloidal Coomassie (#161–0803, Biorad) to visualize protein spots. To determine the proteins’ pI and molecular weight (MW), 2DE SDS-PAGE reference marker proteins (#161–0320 and #161–0378, Bio-Rad) were used. The stained gels were scanned using Alliance 4.7 UVITEC (Cambridge, UK) and the images were analyzed by SameSpots (Cleaver Scientific, UK) software.

### LC-MS/MS

To identify the spotted proteins the following protocol has been performed. At first, the excised spots underwent reduction with Dithiothreitol (DTT), alkylation with Iodoacetamide (IAA), and over-night digestion with Trypsin. The peptides obtained were desalted with StageTip C18. Then, the purified samples were analyzed with nLC-ESI-MS/MS Q Exactive HF, gradient of 33 minutes. The spectra were collected and analyzed using ProteomeDiscoverer 1.4 + Mascot and Scaffold. All MS/MS samples were analyzed using Mascot (Matrix Science, London, UK; version 1.4.1.14). Mascot was set up to search Mascot5_CP_Human_2020_Mammalia (mammals). Mascot was searched with a fragment ion mass tolerance of 20 PPM and a parent ion tolerance of 10.0 PPM. Carbamidomethyl of cysteine was specified in Mascot as a fixed modification. Oxidation of methionine and acetyl of the N-terminus were specified in Mascot as variable modifications. Regarding the criteria for protein identification, Scaffold (version Scaffold_5.3.0, Proteome Software Inc., Portland, OR) was used to validate MS/MS-based peptide and protein identifications. To obtain a strict identification of proteins on Scaffold the identified proteins were filtered using 95% of Peptide Threshold, 99% of Protein Threshold, and a Minimum Number of Peptides of 3. Peptide identifications were accepted if they could be established at greater than 95,0% probability. Peptide Probabilities from Mascot were assigned by the Scaffold Local FDR algorithm and the Peptide Prophet algorithm [17] with Scaffold delta-mass correction. Protein identifications were accepted if they could be established at greater than 99,0% probability and contained at least 3 identified peptides. Protein probabilities were assigned by the Protein Prophet algorithm [18]. Proteins that contained similar peptides and could not be differentiated based on MS/MS analysis alone were grouped to satisfy the principles of parsimony. Proteins sharing significant peptide evidence were grouped into clusters.

### Gene Ontology analysis

Gene ontology (GO) analysis was carried out using ClueGO (v2.5.8) [19] and CluePedia (v1.5.8) running in Cytoscape (v3.10.1), analyzing the protein involvement and the protein-protein interaction for Biological Process, Cellular Component, and Pathway. UniProt accession numbers were used as input data and evaluated against the entire ontology term set using homo sapiens annotation. Ontology term enrichment schematics were generated using Cytoscape [20].

### Western blotting

Proteins were extracted using RIPA buffer and then the protein concentration was evaluated using a BCA kit. Protein lysates (20 μg) were separated on precast 8–12% SDS-polyacrylamide gel using MES or MOPS buffer and electroblotted onto polyvinyl difluoride membrane (PVDF; Sigma-Aldrich). Nonspecific binding sites were blocked in Blocking Buffer (Thermo, USA) for 15 min at RT. Membranes were then incubated overnight at 4°C with the following primary antibodies, diluted in the blocking solution: rabbit anti-GSTP1 (1:10000, ab138491, Abcam), rabbit anti-vinculin (1:1000 Abcam, ab219649, UK), and rabbit anti-GAPDH (PA1–987-HRP, ThermoFisher Scientific). As secondary antibodies, peroxidase-conjugated anti-rabbit (1:50000, G21234, ThermoFisher Scientific) was used. Immunoreactive bands were visualized by Pierce ECL Substrate (ThermoFisher Scientific), according to the manufacturer’s instructions. The relative densities of immunoreactive bands were determined and normalized upon Vinculin or GAPDH, using ImageJ software [21]. Values were given as RU.

### Statistical analysis

All data were presented as mean ± SEM. Western Blot data analyses were performed using GraphPad Prism 8 (GraphPad Software Inc., San Diego, CA, USA). 2DE spots analysis was performed using Samespots (Cleaver Scientific, UK). Multiple comparisons one-way analysis of variance (ANOVA) followed by Tukey post-hoc test was used. Gene ontology term enrichment was calculated using the ClueGO plug-in [19] with the implementation of a hypergeometric model (Fisher’s exact test) and Bonferroni step-down correction to account for multiple testing. The datasets used for this work are published on Zenodo (https://doi.org/10.5281/zenodo.14979855).

## Results

### Proteomic analyses

**Fig 1:**
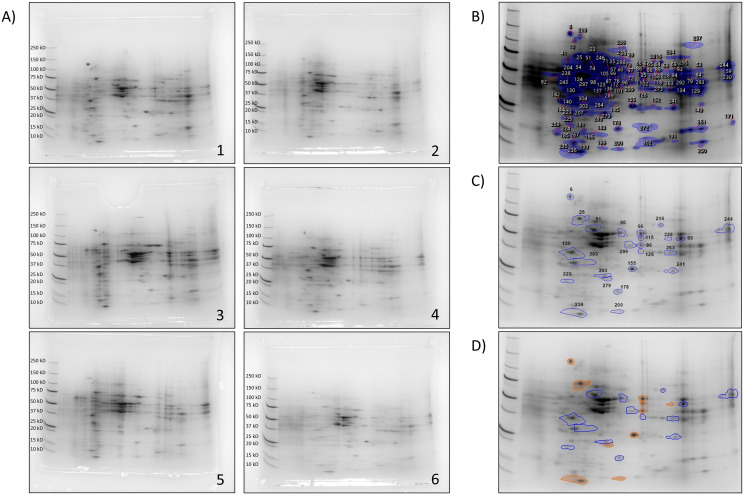
2*DE gels.* **A)** Representative images of two-dimensional electrophoresis gels in ethanol-injured NCI-N87 treated 72 hours with different treatments. 1) CTR, 2) ETOH, 3) ETOH+GABA, 4) ETOH+KLS, 5) ETOH+KLS+GABA, 6) ETOH+KLS-GABA. **B)** Reference image with numbered relevant spots (N = 3); **C)** Representative image with filtered spots. **D)** Filtered spots: orange spots showed statistical significance with p-value ≤ 0.05.

We performed 2DE with protein extracts of NCI-N87 cells injured with ethanol, and subsequently treated with KLS-GABA co-crystal in comparison with KLS and GABA alone or in combination as in the multimodal approach, in order to identify occurred proteomic variations. The 2DE gels-specific spot maps described extremely resolved protein patterns (Fig 1A) that allowed us to co-localize a total of 117 spots during the Samespots image analysis (Fig 1B). Each spot of a reference image was overlapped with the same spot localized on other gel images, obtaining an average of normalized spot volume and an index of intensity fold in respect of the reference image (Fig 1B), as shown in the S1 Table. To highlight the most representative spots, they were subsequently filtered (Fig 1C), as reported in S2 Table, ten of which showed statistical significance (p < 0.05) (Fig 1D). Due to the interesting pattern observed, to characterize the involved proteins, the 24 representative spots were excised for mass spectrometry analysis. A total of 414 non-redundant proteins were identified in 21 spots with 0.00% of False Discovery Rate (S3 Table). Among all the analyzed spots, 3 spots (n = 130, 200, 236) showed no proteins at all at the cited filtering conditions. Interestingly, mass spectrometer and Scaffold’s analyses showed the presence of single proteins in 3 of the 24 excised spots: Protein Disulfide-Isomerase A3 in spot n.60 (PDIA3; P30101), Calcyphosin in spot n.223 (CAPS; Q13938-4), and Glutathione S-transferase P1 in spot n.283 (GSTP1; P09211). Their role in this study will be discussed later in this section.

### Gene Ontology enrichment analyses

**Fig 2:**
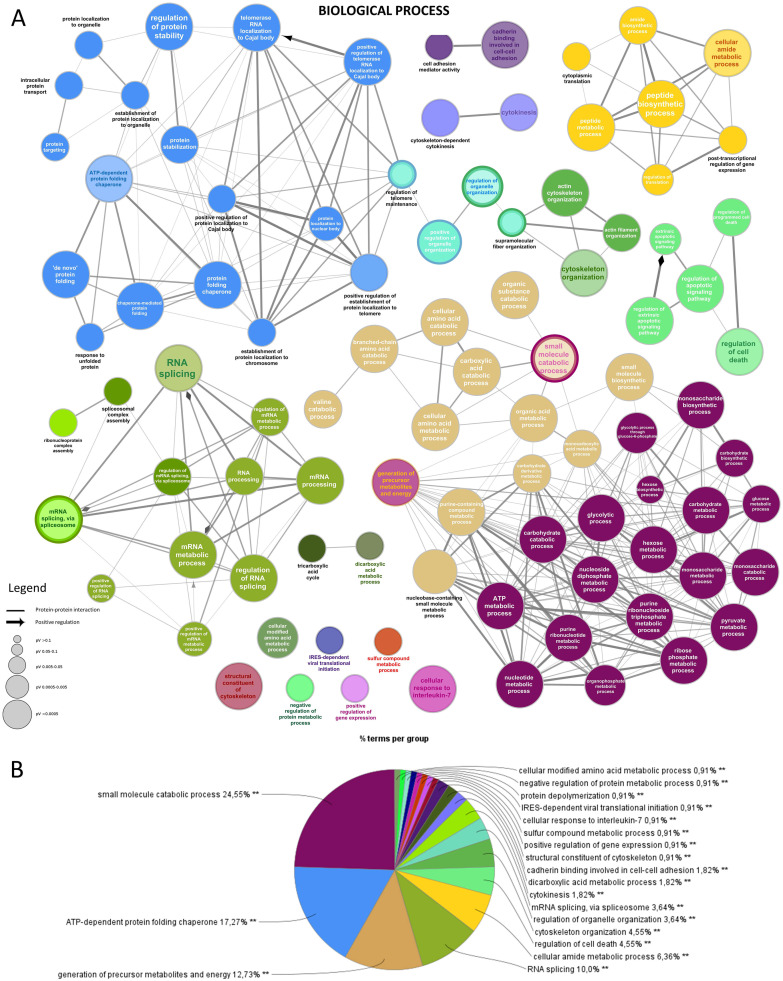
Gene Ontology (GO) enriched analysis on the identified proteins for Biological Process terms. **A)** Functionally grouped network with terms as nodes linked based on their kappa score level (≥0.4), where only the label of the most significant terms per each group appears colored. The node size represents the term enrichment significance, and the edges reflect the correlation weight. Functionally related groups are linked. The GO with a corrected p-value ≤0.05 is considered significant; **B)** Overview chart with functional groups (**p ≤ 0.001).

The identified proteins were classified according to biological processes, cellular components, and pathway groups in which these proteins are involved, observing the protein-protein interaction score (kappa score≥0.4) for each group. Functionally grouped networks were produced for each Gene Ontology group to show scores of group-specific protein-protein interactions for all the identified proteins. The statistically significant (p ≤ 0.05) enrichment terms for the biological process Gene Ontology group produced a network formed by a total of 94 nodes and 356 edges (Fig 2A). The relative percentage (%) of each matched gene, with all functional groups/biological process terms are reported in S1 Fig. The most abundant cluster of proteins was comprised in groups such as “small molecule catabolic process” (24,55%), “ATP-dependent protein folding” (17,27%), and “generation of precursor metabolites and energy” (12,73%). Other abundant groups were “RNA splicing” (10.00%), “cellular amid metabolic process” (6,36%), “regulation of cell death” (4,55%), “cytoskeleton organization” (4,55%), “regulation of organelle organization” (3,64%) “mRNA splicing via spliceosome” (3,64%), and “cadherin binding involved in cell-cell adhesion” (1,82%). Levels of protein-protein interaction were found to be consistent with the percentage of terms per each group. A detailed report of all the biological process groups obtained can be observed in Fig 2B.

**Fig 3:**
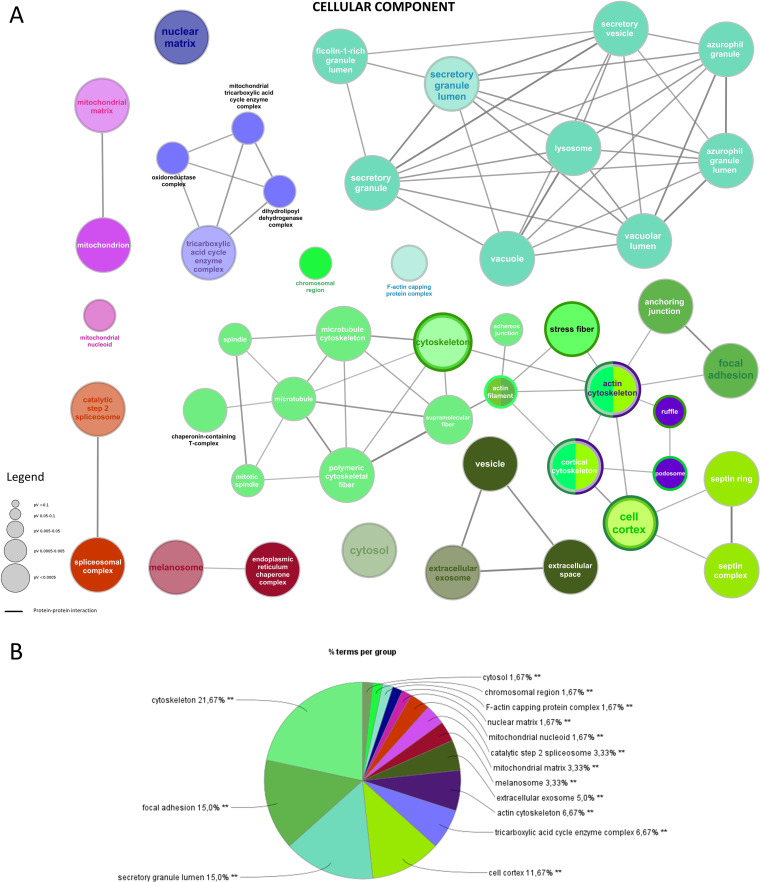
Gene Ontology (GO) enriched analysis on the identified proteins for Cellular Component terms. **A)** Functionally grouped network with terms as nodes linked based on their kappa score level (≥0.4), where only the label of the most significant terms per each group appears colored. The node size represents the term enrichment significance, and the edges reflect the correlation weight. Functionally related groups are linked. The GO with a corrected p-value ≤0.05 is considered significant; **B)** Overview chart with functional groups (**p ≤ 0.001).

Regarding the cellular component group, the statistically significant (p ≤ 0.05) enrichment terms produced a network formed by a total of 47 nodes and 79 edges (Fig 3A), with a major level of protein-protein interaction observed for the “secretory granule lumen” group. The relative percentage (%) of each matched gene, with all functional groups/cellular component terms are reported in S2 Fig. Among all the identified proteins, 21.67% of the total count belonged to the “cytoskeleton” cellular component, while “focal adhesion” and “secretory granule lumen” shared 15,00% of each of the remaining proteins. The 11,67% were represented by proteins of the “cell cortex” and the 6,67% of the “tricarboxylic acid cycle enzyme complex”. Other cellular component groups were “actin cytoskeleton” (6,67%), “extracellular exosome” (5,00%), “melanosome” (3,33%), “mitochondrial matrix” (3,33%), and “catalytic step 2 spliceosome” (3,33%). A detailed report of all the cellular component groups obtained can be observed in Fig 3B.

**Fig 4:**
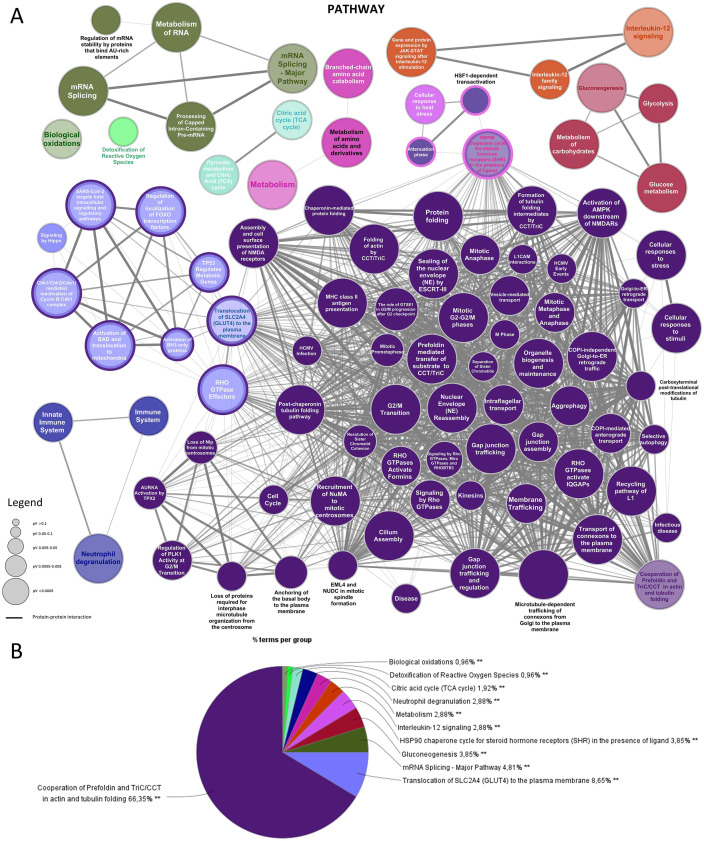
Gene Ontology (GO) enriched analysis on the identified proteins for Pathway terms. **A)** Functionally grouped network with terms as nodes linked based on their kappa score level (≥0.4), where only the label of the most significant terms per each group appears colored. The node size represents the term enrichment significance, and the edges reflect the correlation weight. Functionally related groups are linked. The GO with a corrected p-value ≤0.05 is considered significant; **B)** Overview chart with functional groups (**p ≤ 0.001).

Concerning pathway group, all the statistically significant (p ≤ 0.05) enrichment terms identified were organized into a functional network that showed the major protein-protein interaction level in the “cooperation of prefoldin and Tric/CCT in actin and tubulin folding“group (Fig 4A). The relative percentage (%) of each matched gene, with all functional groups/pathway terms, are reported in S3 Fig. Consistently with the network, the majority of all the proteins analysed clustered in the “cooperation of prefoldin and Tric/CCT in actin and tubulin folding“ group (66,35%). Other relevant groups observed were “translocation of SLC2A4 (GLUT4) to the plasma membrane“ (8,65%), and “mRNA splicing major pathway“ (4,91%). “Gluconeogenesis“ and “HSP90 chaperone cycle for steroid hormone receptors (SHR) in the presence of ligand” shared the 3,85% each of all the remaining proteins, while “interleukin-12 signalling”, “metabolism”, and “neutrophil degranulation” shared the 2,88% each. Other pathways have been observed with a lower percentage of matched identified proteins. A detailed report of all the pathway groups obtained is reported in Fig 4B.

### PDIA3, CAPS, and GSTP1

**Fig 5:**
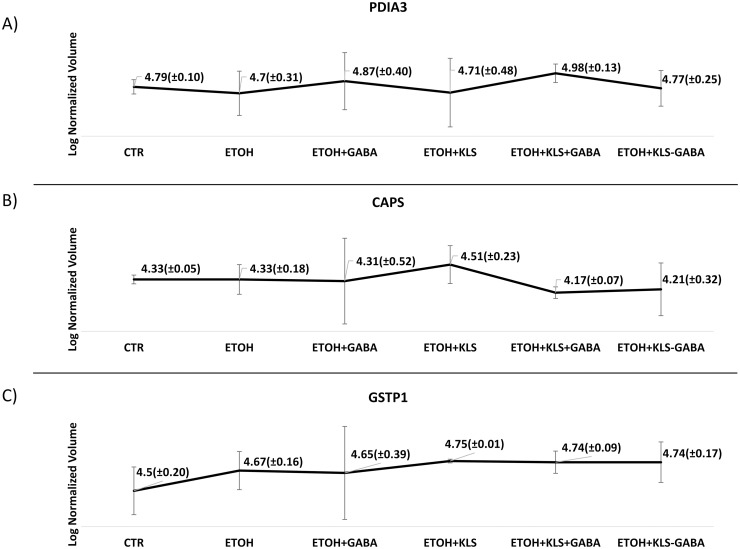
Proteomic results of 2DE of protein extracts of N87 cells. **A)** Differential expression of PDIA3 in 2DE spot n.60 in ethanol-injured N87 treated 72 hours with different treatments; **B)** Differential expression of CAPS in 2DE spot n.223 in ethanol-injured N87 treated 72 hours with different treatments; **C)** Differential expression of GSTP1 in 2DE spot n.283 in ethanol-injured N87 treated 72 hours with different treatments. Data are mean ± SEM. N = 3 (ANOVA one-way).

Among all the visualized spots, three of them showed a single protein each: PDIA3 (spot n.60), CAPS (spot n.223), and GSTP1 (spot n.283). PDIA3 is a multifunctional thiol oxidoreductase involved in the re-folding of misfolded proteins, in the regulation of the folding of newly synthesized glycoproteins, and in the Endoplasmic Reticulum stress [22]. Moreover, PDIA3 appears to be involved in several cellular processes depending on its localization, influencing a broad range of physiological activities. It can be a membrane receptor for Vitamin D metabolites [23], but it can also interact directly with DNA in the nucleus, often promoting the activity of the signal transducer and activator of the transcription 3 (STAT3) complex [24]. These findings correlate with the enrichment analysis reported in this study (Figs 2–4). Expression levels of PDIA3 have been found in *in vivo* and *in vitro* models of gastric cancer [25]. As reported in Fig 5A, the differential expression analysis showed a non-significant increment of PDIA3 protein levels in ethanol-injured NCI-N87 cells after GABA and KLS+GABA administration. Evidence regarding increment of chaperone activity levels with ongoing Endoplasmic Reticulum stress let us to hypothesize the involvement of a PDIA3 in the stress response due to gastric toxicity exerted by GABA and KLS+GABA [26–28]. Interestingly, KLS alone and KLS-GABA co-crystal administration did not cause PDIA3 modulations at all. CAPS is a calcium-binding protein that can be phosphorylated by Protein Kinase A and has been proposed to be involved in the cross signalling between cAMP cascades and calcium-phosphatidylinositol [29,30]. Although its functions are not yet properly uncovered, CAPS has been extensively studied in the last decades in the context of numerous diseases, among which several types of carcinomas, observing changes in CAPS level [31–34]. To date, CAPS has been found expressed in many organs, among which the stomach [35]. Moreover, CAPS seems to play a role in stimulating the proliferation of cancer cells through the modulation of the cited pathways in which CAPS is involved or downregulating cell differentiation following the stimulus of the epidermal growth factor [35,36]. In our 2DE analysis, we found CAPS levels increased in ethanol-injured NCI-N87 cells after KLS administration and slightly decreased after KLS+GABA administration. No changes in CAPS levels were observed for the other conditions (Fig 5B). However, considering that no further details regarding CAPS can be found in the literature that relate to the aim of this study and that no changes were observed following co-crystal administration, the role of CAPS and its modulation in our model under the cited conditions will be no further dissected. Finally, GSTP1 is known to play a relevant role in oxidative stress, detoxifying oxidative stress products, and therapeutic drugs, but also preventing the oxidative degeneration of the gastric mucosa [37,38]. Among the pathways explored in our enrichment analysis “detoxification of reactive oxygen species” and “biological oxidations” were present, although less representative (0.96% each) (Fig 4). Our findings were consistent with results shown in already published studies, where proteomic changes were observed via western blotting [9,11]. For instance, the oxidative stress in NCI-N87 challenged with ETOH has been observed compared to CTR (S4 Fig). In particular, in Western blotting results GABA, KLS, KLS+GABA, and the KLS-GABA cocrystal treatment were able to counteract at different levels the toxic effects of ETOH both at the oxidative stress (4-HNE; CATALASE; SOD1) and inflammatory levels (p-p38; NFkB; p-ERK 1–2; IkBα,PPARγ) [9]. Notably, GSTP1 role in this study has been hypothesized starting from our 2DE findings. As shown in Fig 5C, in our *in vitro* model upon all the tested conditions, there is an increased trend (no statistically significant) of GSTP1 levels compared to the CTR.

**Fig 6:**
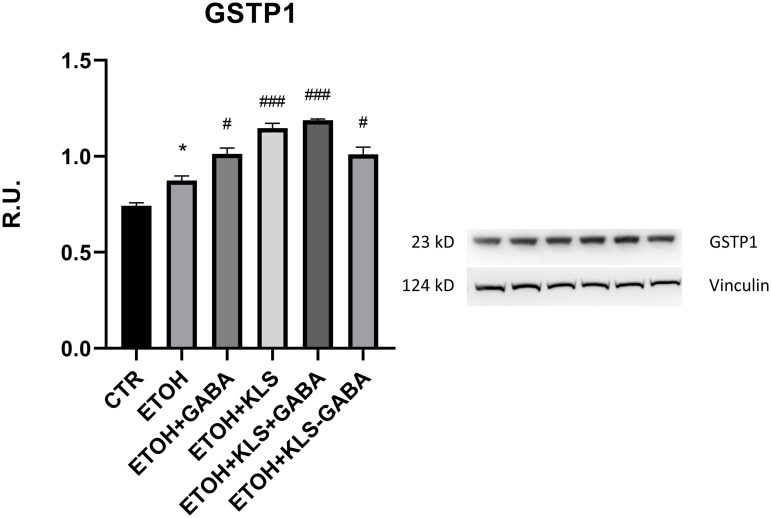
Western Blot analysis of protein extracts of N87 cells. Protein levels of GSTP1 in ethanol-injured NCI-N87 treated 72 hours with different treatments. Data are mean ± SEM. N = 3 (ANOVA one-way, *p ≤ 0.05 vs CTR; ^#^p ≤ 0.05, ^###^p ≤ 0.0005 vs ETOH).

Notably, Western blotting results regarding GSTP1 in the same model and conditions allowed us to clarify the differential expression analysis observed with 2DE regarding this protein. As shown in Fig 6, the protein levels of GSTP1 increased in NCI-N87 cells upon GABA, KLS, and KLS+GABA treatment. Interestingly, GSTP1 levels were remarkably lower after the co-crystal treatment, with a statistically significant decrease compared to the result observed with the 2DE, suggesting a reduction of oxidative stress levels in the gastric epithelium model due to a presumably higher gastro-tolerability of the co-crystal drug compared to the other drugs.

## Discussion

With the aim to evaluate proteomic changes in the ethanol-injured gastric epithelium model following the administration of KLS, GABA, their combination, and the co-crystal, we performed a 2DE-based differential expression analysis followed by LC-MS/MS and Gene Ontology-based enrichment analysis. As previously shown in other studies, NSAIDs treatment can be linked to an increased risk of gastric erosion and ulceration, effects that have been found counteracted in GABA-based combined therapy [39,40]. Our enrichment analysis showed the clustering of many identified proteins in Gene Ontology groups related to different types of cell-cell junctions, among all “cadherin binding involved in cell-cell adhesion” (Fig 2). Further analyses revealed the modulation of several genes related to the effects of KLS and gabapentin combination therapy in the same model used in this study [11,12,40]. Interestingly, the damage suffered by the gastric epithelium under ethanol injury seemed to be derived from oxidative and inflammatory processes. KLS alone did not affect the oxidative stress caused by the ethanol but exerted only inflammatory effects reducing NFkB levels, while GABA alone and the combined therapy exerted positive effects, downregulating toxic genes (NFkB, p-38, p-ERK 1–2) and upregulating protective ones (CCK, MUC5B, GALNTN8, CLDN5, GST). KLS-GABA co-crystal showed dramatically reduced gastrointestinal side effects that are characteristic of NSAIDs, strengthening their typical therapeutic effects [9]. These findings match with the results showed in this study by 2DE and enrichments analysis (Figs 2–4). For instance, among the three individually spotted proteins PDIA3 is known to be related to p38/MAPK pathway by which PDIA can be activated in order to exert anti-oxidant and anti-Endoplasmic Reticulum stress effects [41]. As showed in the results section (Fig 5A), we found reduced levels of PDIA3 protein after KLS and KLS-GABA co-crystal treatment in the tested model that can be related to a reduced overall gastric stress linked to the improved gastro-tolerability of the co-crystal drug, as already said, but also to reduced NFkB stimulus. However, further analyses are needed to better understand this correlation. Regarding the oxidative stress, we showed the presence of Gene Ontology groups and related terms that match with the recent literature (Fig 4). The novelty is indeed represented by the findings confirmed by Western blotting related to GSTP1 (Figs 5C and 6) and its reduced level in the cells treated with KLS-GABA co-crystal, expanding the knowledge around factors and mechanisms involved in the co-crystal effects.

2DE is a powerful technique for protein separation. However, 2DE shows several challenges in the proteome characterization. Among the known limitations of this technique, it is possible to find low sensitivity for scarce or hydrophobic proteins, such as membrane-bound receptors, and difficulty in resolving post-translationally modified isoforms, which are often crucial in signaling cascades. Consequently, 2DE-based proteomics may underrepresent critical regulators involved in complex biological processes. As observed in our results, co-migration of multiple proteins to single spots occurred at almost each spot (S3 Table). Also, proteins potentially migrated differently due to post-translational processing and fragmentation. Due to these reasons, we chose to investigate for the proteomic differential expression only the 3 proteins found to be alone in three different spots after data analysis with Scaffold. Nonetheless, the modulation of the 24 filtered spots under each condition let us to proceed further with mass spectrometry analysis and Gene Ontology-based enrichment analyses in order to observe what lies behind the 2DE results. The following analyses showed to us the presence of relevant proteins and their interaction in the gastric epithelium model under the cited conditions, demonstrating the reliability of the chosen proteomic approach in our context in order to clarify the exact mechanism underlying the striking efficacy enhancement associated with the KLS-GABA co-crystal administration.

Our findings align with emerging evidence that co-crystals can shape early pharmacological responses through solvation dynamics and transient non-equilibrium states. Specifically, the KLS-GABA co-crystal exhibited enhanced gastrointestinal permeability and systemic distribution. These physicochemical characteristics may help refine drug delivery strategies and improve therapeutic impact, underscoring the potential of co-crystal formulations in clinical applications.

## Conclusion

The present study represents a starting point for the improvement of model characterization in methods and analyses. Nevertheless, to the best of our knowledge, the present investigation is the first to report gel-based differential proteome profiling in an *in vitro* model of human leaky gut for gastro-tolerability evaluation of drugs. In conclusion, these data collectively confirm the proposed KLS-GABA co-crystal gastro-tolerability in an *in vitro* model of gastric epithelium under ethanol injury, showing a wide view of proteomic changes that occur after the treatment with the cited drugs, highlighting the clinical reliability of KLS-GABA co-crystal in context like chronic pain.

## Supporting information

S1 Table Overview of all the 117 identified spots. One-way Anova score, average of normalized spot volume and an index of intensity fold in respect of the reference image are reported in the table. Data are mean ± SEM. N = 3. Statistical significance was considered for spots with p-value ≤ 0.05.
(DOCX)

S2 Table Overview of the 24 differentially expressed excised spots. One-way Anova score, average of normalized spot volume and an index of intensity fold in respect of the reference image are reported in the table. Data are mean ± SEM. N = 3. Statistical significance was considered for spots with p-value ≤ 0.05.
(DOCX)

S3 Table List of identified proteins confidently assigned to 2DE gel spots. Of all the 24 excised spots, 3 spots showed no proteins at all (spots n# 130; 200; 236).
(DOCX)

S1 Fig Gene Ontology (GO) enriched analysis on the identified proteins for Biological Process terms. GO/Biological Process terms specific for representative genes. The bars represent the number of genes associated with the terms. The percentage of genes per term is shown as bar label (*p ≤ 0.05; **p < 0.001).
(DOCX)

S2 Fig Gene Ontology (GO) enriched analysis on the identified proteins for Cellular Component terms. GO/Cellular Component terms specific for representative genes. The bars represent the number of genes associated with the terms. The percentage of genes per term is shown as bar label (*p ≤ 0.05; **p < 0.001).
(DOCX)

S3 Fig Gene Ontology (GO) enriched analysis on the identified proteins for Pathway terms. GO/Pathway terms specific for representative genes. The bars represent the number of genes associated with the terms. The percentage of genes per term is shown as bar label (*p ≤ 0.05; **p < 0.001).
(DOCX)

S4 Fig Effects of drug treatments in the leaky gut *in vitro* model. Representative bright field pictures of untreated control N87 and ethanol-injured N87 treated 72 hours with different treatments. Bar = 400 μm.
(DOCX)

S5 Fig Raw_images. Figure 1. Raw images of 2DE gels divided by replicates. For Figure 1 panel the first replicate was used.
(PDF)

S6 Fig Raw_images. Figure 3.2. Raw images of Western Blotting analysis for GSTP1. On the left images, the highlighted sections show bands for GSTP1. On the right, the highlighted sections show the reference on which GSTP1 data were normalized. The panel in figure 3.2 in the manuscript shows sections of the figure here highlighted in a green square.
(PDF)

S7 Fig Raw_images. Figure S7. Effects of drug treatments in the leaky gut in vitro model. Representative bright field pictures of untreated control N87 and ethanol-injured N87 treated 72 hours with different treatments. Bar = 400 μm.
(PDF)

S1 Data Sheet 1. Peptide Report. Sheet 2. Protein Report. (XLSX)
