# Supplementary material for: Evaluation of the gastrotolerability of ketoprofen, lysine, and gabapentin co-crystal administration in an in vitro model of gastric epithelium: a proteomic update
Source: PLoS One. 2025 Jul 29;20(7):e0328496. doi: 10.1371/journal.pone.0328496 (PMC12306739; doi:10.1371/journal.pone.0328496)
Supplement: S1 Table — One-way Anova score, average of normalized spot volume and an index of intensity fold in respect of the reference image are reported in the table. Data are mean ± SEM. N = 3. Statistical significance was considered for spots with p-value ≤ 0.05. (DOCX) [file pone.0328496.s001.docx]

**S1 Table.** **Overview of all the 117 identified spots.** One-way Anova score, average of normalized spot volume and an index of intensity fold in respect of the reference image are reported in the table. Data are mean ± SEM. N=3. Statistical significance was considered for spots with p-value ≤ 0.05.

| # | Anova (p) | Fold | Average Normalized Volumes | | | | | |
| --- | --- | --- | --- | --- | --- | --- | --- | --- |
|  |  |  | **CTR** | **ETOH** | **GABA** | **KLS** | **KLS+GABA** | **KLS/GABA** |
| 66 | 1,18E-04 | 4,2 | 5,45E+04 | 3,71E+04 | 6,58E+04 | 1,55E+04 | 2,98E+04 | 4,43E+04 |
| 204 | 1,34E-04 | 1,6 | 9,04E+04 | 1,30E+05 | 8,68E+04 | 1,18E+05 | 1,42E+05 | 9,12E+04 |
| 102 | 3,92E-04 | 5,3 | 3248,671 | 1766,027 | 5599,411 | 1047,816 | 2428,731 | 2291,819 |
| 86 | 0,001 | 5,2 | 1,99E+04 | 1,33E+04 | 3,33E+04 | 6338,626 | 1,86E+04 | 1,62E+04 |
| 115 | 0,002 | 3,6 | 2,46E+04 | 1,94E+04 | 3,72E+04 | 1,03E+04 | 1,77E+04 | 2,26E+04 |
| 87 | 0,002 | 2,4 | 2,68E+04 | 4,50E+04 | 2,08E+04 | 4,34E+04 | 3,48E+04 | 5,01E+04 |
| 297 | 0,003 | 1,4 | 3,08E+05 | 3,75E+05 | 3,10E+05 | 3,53E+05 | 3,93E+05 | 4,22E+05 |
| 71 | 0,005 | 1,6 | 6,89E+04 | 9,40E+04 | 6,11E+04 | 9,41E+04 | 8,76E+04 | 9,74E+04 |
| 155 | 0,005 | 2,4 | 6,17E+04 | 2,58E+04 | 6,04E+04 | 5,12E+04 | 4,80E+04 | 5,71E+04 |
| 41 | 0,006 | 2,5 | 5,01E+04 | 8,02E+04 | 1,26E+05 | 5,89E+04 | 8,72E+04 | 6,33E+04 |
| 215 | 0,006 | 2,5 | 4,53E+04 | 5,32E+04 | 9,22E+04 | 4,52E+04 | 1,14E+05 | 4,53E+04 |
| 25 | 0,013 | 1,6 | 1,29E+05 | 1,70E+05 | 1,93E+05 | 1,42E+05 | 2,09E+05 | 1,62E+05 |
| 289 | 0,019 | 1,8 | 7,70E+04 | 1,33E+05 | 1,38E+05 | 1,08E+05 | 1,26E+05 | 1,35E+05 |
| 6 | 0,022 | 2,1 | 4,45E+04 | 2,66E+04 | 2,61E+04 | 2,75E+04 | 2,43E+04 | 2,15E+04 |
| 236 | 0,022 | 2 | 1,25E+05 | 1,39E+05 | 1,03E+05 | 1,56E+05 | 1,17E+05 | 7,92E+04 |
| 142 | 0,022 | 3,2 | 1,22E+04 | 3843,624 | 4020,881 | 6500,721 | 3906,389 | 5897,083 |
| 54 | 0,023 | 1,8 | 5,30E+04 | 7,19E+04 | 7,56E+04 | 7,82E+04 | 9,61E+04 | 8,18E+04 |
| 144 | 0,029 | 2,7 | 1,40E+04 | 7323,429 | 5544,739 | 9530,505 | 5207,667 | 8970,385 |
| 200 | 0,03 | 1,6 | 1,61E+04 | 1,84E+04 | 1,48E+04 | 1,85E+04 | 2,41E+04 | 1,95E+04 |
| 34 | 0,032 | 2,9 | 1,96E+04 | 1,90E+04 | 5,49E+04 | 1,90E+04 | 4,10E+04 | 3,16E+04 |
| 279 | 0,036 | 1,4 | 2,22E+04 | 2,36E+04 | 2,40E+04 | 3,17E+04 | 2,86E+04 | 3,12E+04 |
| 228 | 0,042 | 2,3 | 8261,461 | 1,66E+04 | 7341,488 | 9796,688 | 1,14E+04 | 7453,534 |
| 134 | 0,056 | 3,7 | 5,25E+04 | 3,21E+04 | 6,23E+04 | 1,12E+05 | 3,00E+04 | 3,91E+04 |
| 76 | 0,057 | 2,5 | 2,69E+04 | 3,59E+04 | 4,86E+04 | 3,87E+04 | 6,67E+04 | 3,43E+04 |
| 136 | 0,059 | 1,9 | 2,99E+04 | 3,39E+04 | 4,20E+04 | 3,51E+04 | 3,77E+04 | 5,57E+04 |
| 97 | 0,061 | 8,3 | 4859,249 | 6059,79 | 2,48E+04 | 3759,211 | 8197,439 | 2997,056 |
| 145 | 0,062 | 2,8 | 4445,801 | 4514,57 | 3665,15 | 8205,499 | 2942,204 | 5064,674 |
| 72 | 0,063 | 6 | 1,41E+04 | 1,97E+04 | 6,12E+04 | 1,26E+04 | 1,78E+04 | 1,03E+04 |
| 112 | 0,063 | 2,1 | 2,49E+04 | 2,68E+04 | 4,92E+04 | 2,52E+04 | 3,63E+04 | 2,33E+04 |
| 292 | 0,064 | 2,1 | 1,73E+05 | 1,36E+05 | 2,07E+05 | 2,81E+05 | 1,32E+05 | 1,61E+05 |
| 207 | 0,074 | 1,8 | 1,23E+05 | 1,02E+05 | 1,81E+05 | 1,33E+05 | 1,63E+05 | 1,31E+05 |
| 29 | 0,08 | 4,3 | 1996,794 | 2031,66 | 8631,024 | 2256,936 | 2765,885 | 3476,152 |
| 299 | 0,083 | 2,7 | 4,64E+04 | 4,49E+04 | 1,22E+05 | 5,26E+04 | 6,70E+04 | 4,52E+04 |
| 105 | 0,094 | 2,1 | 2,80E+04 | 3,17E+04 | 1,73E+04 | 3,57E+04 | 2,05E+04 | 3,68E+04 |
| 35 | 0,109 | 2,1 | 1,74E+04 | 2,35E+04 | 3,70E+04 | 1,73E+04 | 3,40E+04 | 2,03E+04 |
| 88 | 0,112 | 2,2 | 9072,417 | 9709,192 | 5525,018 | 4431,331 | 6628,03 | 8708,035 |
| 298 | 0,116 | 2,6 | 6353,979 | 6286,636 | 7378,547 | 3640,442 | 9421,752 | 4436,893 |
| 123 | 0,12 | 1,4 | 2,45E+05 | 2,59E+05 | 2,06E+05 | 2,93E+05 | 2,51E+05 | 2,46E+05 |
| 69 | 0,12 | 3,8 | 5,87E+04 | 4,31E+04 | 3,89E+04 | 7,23E+04 | 1,89E+04 | 3,43E+04 |
| 293 | 0,12 | 2,7 | 1,70E+05 | 1,44E+05 | 6,17E+04 | 1,14E+05 | 1,57E+05 | 1,15E+05 |
| 73 | 0,123 | 3,8 | 1,97E+04 | 1,18E+04 | 4,48E+04 | 1,71E+04 | 1,53E+04 | 1,41E+04 |
| 74 | 0,132 | 1,6 | 3,39E+05 | 3,44E+05 | 2,22E+05 | 3,34E+05 | 2,62E+05 | 3,54E+05 |
| 51 | 0,14 | 1,3 | 1,24E+05 | 1,25E+05 | 1,07E+05 | 1,23E+05 | 1,13E+05 | 1,34E+05 |
| 53 | 0,146 | 1,9 | 7,70E+04 | 7,95E+04 | 4,81E+04 | 8,91E+04 | 5,49E+04 | 6,81E+04 |
| 126 | 0,151 | 2,3 | 1,54E+04 | 1,61E+04 | 2,45E+04 | 1,07E+04 | 1,81E+04 | 1,19E+04 |
| 283 | 0,169 | 1,7 | 3,26E+04 | 4,72E+04 | 4,87E+04 | 5,61E+04 | 5,57E+04 | 5,62E+04 |
| 124 | 0,174 | 1,5 | 3,84E+04 | 2,94E+04 | 4,45E+04 | 4,48E+04 | 4,44E+04 | 3,35E+04 |
| 59 | 0,178 | 1,7 | 3,93E+04 | 3,76E+04 | 3,73E+04 | 3,61E+04 | 4,39E+04 | 6,05E+04 |
| 79 | 0,181 | 2 | 2,40E+04 | 3,57E+04 | 2,13E+04 | 4,36E+04 | 2,53E+04 | 3,29E+04 |
| 238 | 0,188 | 1,8 | 2,88E+04 | 5,07E+04 | 3,15E+04 | 4,07E+04 | 2,81E+04 | 3,81E+04 |
| 38 | 0,202 | 2,3 | 8649,139 | 4343,074 | 3827,172 | 5265,665 | 4894,394 | 5593,638 |
| 223 | 0,216 | 2,3 | 2,16E+04 | 2,17E+04 | 2,32E+04 | 3,35E+04 | 1,47E+04 | 1,71E+04 |
| 149 | 0,222 | 1,7 | 7012,328 | 6679,718 | 5717,025 | 4059,941 | 5972,188 | 4087,89 |
| 84 | 0,229 | 2,6 | 7872,473 | 8168,445 | 3097,426 | 5065,324 | 7338,354 | 4472,881 |
| 219 | 0,236 | 1,8 | 8537,819 | 6069,515 | 8713,917 | 1,05E+04 | 5740,645 | 6458,645 |
| 230 | 0,245 | 1,7 | 1,02E+05 | 9,39E+04 | 8,95E+04 | 9,72E+04 | 1,12E+05 | 1,51E+05 |
| 304 | 0,26 | 1,8 | 5,56E+04 | 5,23E+04 | 9,34E+04 | 6,27E+04 | 8,29E+04 | 7,88E+04 |
| 178 | 0,273 | 1,5 | 3,17E+04 | 2,72E+04 | 2,34E+04 | 3,47E+04 | 3,45E+04 | 3,44E+04 |
| 98 | 0,275 | 1,6 | 1,00E+05 | 7,88E+04 | 6,41E+04 | 1,02E+05 | 9,50E+04 | 8,31E+04 |
| 273 | 0,285 | 1,8 | 9,62E+04 | 8,58E+04 | 6,89E+04 | 8,44E+04 | 8,17E+04 | 1,21E+05 |
| 259 | 0,288 | 1,7 | 1,97E+04 | 1,25E+04 | 1,54E+04 | 2,17E+04 | 1,95E+04 | 1,63E+04 |
| 96 | 0,289 | 3 | 5504,325 | 4398,883 | 1,27E+04 | 4578,727 | 5363,707 | 4200,023 |
| 246 | 0,305 | 1,4 | 3,00E+05 | 3,60E+05 | 4,04E+05 | 3,51E+05 | 3,98E+05 | 4,10E+05 |
| 118 | 0,309 | 2,3 | 1,10E+04 | 1,60E+04 | 7025,66 | 1,17E+04 | 9133,854 | 9196,962 |
| 294 | 0,31 | 1,8 | 1,90E+04 | 1,41E+04 | 1,08E+04 | 1,45E+04 | 1,10E+04 | 1,60E+04 |
| 284 | 0,314 | 1,7 | 1,26E+05 | 1,70E+05 | 1,84E+05 | 1,77E+05 | 2,02E+05 | 2,14E+05 |
| 244 | 0,318 | 1,4 | 1,49E+05 | 1,38E+05 | 1,43E+05 | 1,52E+05 | 1,65E+05 | 1,98E+05 |
| 186 | 0,324 | 1,9 | 4833,326 | 4357,847 | 3655,375 | 4246,02 | 6764,89 | 6614,469 |
| 101 | 0,325 | 1,9 | 2,34E+04 | 2,62E+04 | 4,24E+04 | 2,23E+04 | 3,61E+04 | 2,64E+04 |
| 225 | 0,344 | 1,7 | 4796,58 | 4632,048 | 4863,1 | 5389,692 | 4395,098 | 3084,566 |
| 10 | 0,348 | 1,6 | 1,10E+04 | 7236,35 | 7724,431 | 8752,55 | 7511,303 | 6879,5 |
| 23 | 0,407 | 1,5 | 1554,588 | 1939,525 | 1486,738 | 1327,772 | 1901,355 | 1765,138 |
| 93 | 0,41 | 2,3 | 4,06E+04 | 2,54E+04 | 3,15E+04 | 4,88E+04 | 3,38E+04 | 2,11E+04 |
| 99 | 0,429 | 1,7 | 3,61E+04 | 4,89E+04 | 6,15E+04 | 5,62E+04 | 5,72E+04 | 4,93E+04 |
| 288 | 0,433 | 1,7 | 3,94E+04 | 5,70E+04 | 6,04E+04 | 5,55E+04 | 6,88E+04 | 5,44E+04 |
| 183 | 0,441 | 1,8 | 6,94E+04 | 8,26E+04 | 1,10E+05 | 1,13E+05 | 1,25E+05 | 1,28E+05 |
| 60 | 0,446 | 1,8 | 6,26E+04 | 5,31E+04 | 7,99E+04 | 5,80E+04 | 9,57E+04 | 6,09E+04 |
| 193 | 0,509 | 1,8 | 6399,632 | 5566,68 | 8677,316 | 6431,534 | 4808,102 | 5146,547 |
| 171 | 0,519 | 1,6 | 6221,398 | 6048,203 | 6478,236 | 5402,806 | 5393,281 | 8802,4 |
| 58 | 0,528 | 2 | 5571,558 | 9990,407 | 5503,526 | 1,10E+04 | 6589,994 | 9137,962 |
| 64 | 0,532 | 2,1 | 1,01E+04 | 1,20E+04 | 6549,326 | 7749,472 | 5765,492 | 7889,286 |
| 224 | 0,538 | 1,7 | 4626,76 | 4793,445 | 5386,836 | 7808,639 | 4508,212 | 5244,388 |
| 63 | 0,543 | 2 | 2,32E+04 | 1,92E+04 | 1,80E+04 | 2,14E+04 | 1,34E+04 | 1,13E+04 |
| 140 | 0,55 | 1,5 | 8,66E+04 | 7,56E+04 | 9,36E+04 | 1,17E+05 | 1,08E+05 | 8,87E+04 |
| 235 | 0,574 | 1,5 | 4,04E+04 | 3,74E+04 | 3,53E+04 | 5,17E+04 | 4,09E+04 | 3,82E+04 |
| 129 | 0,585 | 1,5 | 2,06E+05 | 2,14E+05 | 1,88E+05 | 2,66E+05 | 2,80E+05 | 2,13E+05 |
| 263 | 0,59 | 1,7 | 4,69E+04 | 3,78E+04 | 3,89E+04 | 5,23E+04 | 3,18E+04 | 5,35E+04 |
| 111 | 0,597 | 2,1 | 1,07E+04 | 8081,001 | 8604,224 | 1,17E+04 | 7350,519 | 5475,492 |
| 57 | 0,601 | 1,6 | 6,65E+04 | 8,53E+04 | 8,70E+04 | 8,56E+04 | 1,08E+05 | 8,44E+04 |
| 261 | 0,619 | 1,5 | 1,59E+05 | 1,22E+05 | 1,13E+05 | 1,27E+05 | 1,36E+05 | 1,08E+05 |
| 152 | 0,643 | 1,3 | 6,19E+04 | 5,69E+04 | 6,51E+04 | 5,42E+04 | 6,98E+04 | 6,92E+04 |
| 181 | 0,647 | 1,5 | 4005,237 | 5875,726 | 4636,046 | 4896,167 | 4524,592 | 3978,748 |
| 130 | 0,677 | 1,3 | 2,30E+05 | 2,29E+05 | 2,89E+05 | 2,18E+05 | 2,42E+05 | 2,32E+05 |
| 116 | 0,679 | 2,4 | 7309,626 | 1,40E+04 | 6401,873 | 5874,458 | 8084,929 | 8108,177 |
| 243 | 0,691 | 1,3 | 5,37E+04 | 6,29E+04 | 4,83E+04 | 5,28E+04 | 4,88E+04 | 5,17E+04 |
| 185 | 0,692 | 1,7 | 1,37E+04 | 1,24E+04 | 1,21E+04 | 1,51E+04 | 8688,815 | 9361,803 |
| 137 | 0,7 | 1,3 | 1,48E+04 | 1,93E+04 | 1,55E+04 | 1,99E+04 | 1,79E+04 | 1,92E+04 |
| 295 | 0,734 | 1,7 | 2,31E+04 | 1,70E+04 | 1,39E+04 | 2,17E+04 | 1,54E+04 | 1,86E+04 |
| 257 | 0,754 | 1,4 | 4,70E+04 | 4,19E+04 | 5,51E+04 | 3,92E+04 | 3,82E+04 | 4,22E+04 |
| 272 | 0,764 | 1,3 | 6,48E+04 | 4,86E+04 | 6,31E+04 | 5,03E+04 | 5,23E+04 | 6,31E+04 |
| 241 | 0,791 | 1,4 | 3,84E+04 | 3,33E+04 | 4,28E+04 | 4,30E+04 | 4,08E+04 | 4,83E+04 |
| 85 | 0,792 | 1,6 | 9516,828 | 7675,937 | 8098,154 | 8343,408 | 8033,29 | 5804,498 |
| 250 | 0,792 | 1,7 | 4,86E+04 | 5,39E+04 | 4,25E+04 | 4,47E+04 | 7,43E+04 | 4,33E+04 |
| 303 | 0,795 | 1,3 | 9,32E+04 | 8,27E+04 | 1,06E+05 | 9,28E+04 | 1,04E+05 | 1,04E+05 |
| 191 | 0,818 | 2,5 | 2404,628 | 5907,079 | 3005,859 | 2974,784 | 4489,812 | 3479,136 |
| 216 | 0,834 | 1,8 | 8596,424 | 7716,122 | 5651,13 | 9520,579 | 7964,847 | 1,00E+04 |
| 89 | 0,863 | 1,4 | 5187,504 | 4709,142 | 4002,497 | 5010,717 | 3616,933 | 3884,473 |
| 94 | 0,864 | 2,1 | 6970,959 | 1,08E+04 | 5301,638 | 6901,744 | 6578,246 | 5112,299 |
| 302 | 0,867 | 1,2 | 1,34E+05 | 1,10E+05 | 1,12E+05 | 1,35E+05 | 1,28E+05 | 1,23E+05 |
| 65 | 0,87 | 2,2 | 1,62E+04 | 2,08E+04 | 9532,551 | 1,19E+04 | 1,13E+04 | 1,43E+04 |
| 68 | 0,874 | 2,1 | 1,24E+04 | 1,92E+04 | 1,53E+04 | 1,81E+04 | 9311,97 | 1,39E+04 |
| 82 | 0,878 | 1,4 | 8,84E+04 | 9,87E+04 | 1,26E+05 | 1,10E+05 | 1,24E+05 | 8,90E+04 |
| 267 | 0,886 | 1,2 | 5,30E+04 | 5,31E+04 | 5,21E+04 | 5,28E+04 | 4,26E+04 | 4,37E+04 |
| 199 | 0,966 | 1,2 | 1,04E+04 | 1,06E+04 | 9965,287 | 1,15E+04 | 1,03E+04 | 1,17E+04 |
