# Supplementary material for: Evaluation of the gastrotolerability of ketoprofen, lysine, and gabapentin co-crystal administration in an in vitro model of gastric epithelium: a proteomic update
Source: PLoS One. 2025 Jul 29;20(7):e0328496. doi: 10.1371/journal.pone.0328496 (PMC12306739; doi:10.1371/journal.pone.0328496)
Supplement: S2 Table — One-way Anova score, average of normalized spot volume and an index of intensity fold in respect of the reference image are reported in the table. Data are mean ± SEM. N = 3. Statistical significance was considered for spots with p-value ≤ 0.05. (DOCX) [file pone.0328496.s002.docx]

**S2 Table.** **Overview of the 24 differentially expressed excised spots.** One-way Anova score, average of normalized spot volume and an index of intensity fold in respect of the reference image are reported in the table. Data are mean ± SEM. N=3. Statistical significance was considered for spots with p-value ≤ 0.05.

| # | Anova (p) | Fold | Average Normalized Volumes | | | | | |
| --- | --- | --- | --- | --- | --- | --- | --- | --- |
|  |  |  | **CTR** | **ETOH** | **GABA** | **KLS** | **KLS+GABA** | **KLS/GABA** |
| 66 | 1,18E-04 | 4,2 | 5,45E+04 | 3,71E+04 | 6,58E+04 | 1,55E+04 | 2,98E+04 | 4,43E+04 |
| 86 | 0,001 | 5,2 | 1,99E+04 | 1,33E+04 | 3,33E+04 | 6338,626 | 1,86E+04 | 1,62E+04 |
| 115 | 0,002 | 3,6 | 2,46E+04 | 1,94E+04 | 3,72E+04 | 1,03E+04 | 1,77E+04 | 2,26E+04 |
| 155 | 0,005 | 2,4 | 6,17E+04 | 2,58E+04 | 6,04E+04 | 5,12E+04 | 4,80E+04 | 5,71E+04 |
| 25 | 0,013 | 1,6 | 1,29E+05 | 1,70E+05 | 1,93E+05 | 1,42E+05 | 2,09E+05 | 1,62E+05 |
| 6 | 0,022 | 2,1 | 4,45E+04 | 2,66E+04 | 2,61E+04 | 2,75E+04 | 2,43E+04 | 2,15E+04 |
| 236 | 0,022 | 2 | 1,25E+05 | 1,39E+05 | 1,03E+05 | 1,56E+05 | 1,17E+05 | 7,92E+04 |
| 200 | 0,03 | 1,6 | 1,61E+04 | 1,84E+04 | 1,48E+04 | 1,85E+04 | 2,41E+04 | 1,95E+04 |
| 279 | 0,036 | 1,4 | 2,22E+04 | 2,36E+04 | 2,40E+04 | 3,17E+04 | 2,86E+04 | 3,12E+04 |
| 228 | 0,042 | 2,3 | 8261,461 | 1,66E+04 | 7341,488 | 9796,688 | 1,14E+04 | 7453,534 |
| 299 | 0,083 | 2,7 | 4,64E+04 | 4,49E+04 | 1,22E+05 | 5,26E+04 | 6,70E+04 | 4,52E+04 |
| 51 | 0,14 | 1,3 | 1,24E+05 | 1,25E+05 | 1,07E+05 | 1,23E+05 | 1,13E+05 | 1,34E+05 |
| 126 | 0,151 | 2,3 | 1,54E+04 | 1,61E+04 | 2,45E+04 | 1,07E+04 | 1,81E+04 | 1,19E+04 |
| 283 | 0,169 | 1,7 | 3,26E+04 | 4,72E+04 | 4,87E+04 | 5,61E+04 | 5,57E+04 | 5,62E+04 |
| 223 | 0,216 | 2,3 | 2,16E+04 | 2,17E+04 | 2,32E+04 | 3,35E+04 | 1,47E+04 | 1,71E+04 |
| 178 | 0,273 | 1,5 | 3,17E+04 | 2,72E+04 | 2,34E+04 | 3,47E+04 | 3,45E+04 | 3,44E+04 |
| 244 | 0,318 | 1,4 | 1,49E+05 | 1,38E+05 | 1,43E+05 | 1,52E+05 | 1,65E+05 | 1,98E+05 |
| 93 | 0,41 | 2,3 | 4,06E+04 | 2,54E+04 | 3,15E+04 | 4,88E+04 | 3,38E+04 | 2,11E+04 |
| 60 | 0,446 | 1,8 | 6,26E+04 | 5,31E+04 | 7,99E+04 | 5,80E+04 | 9,57E+04 | 6,09E+04 |
| 263 | 0,59 | 1,7 | 4,69E+04 | 3,78E+04 | 3,89E+04 | 5,23E+04 | 3,18E+04 | 5,35E+04 |
| 130 | 0,677 | 1,3 | 2,30E+05 | 2,29E+05 | 2,89E+05 | 2,18E+05 | 2,42E+05 | 2,32E+05 |
| 241 | 0,791 | 1,4 | 3,84E+04 | 3,33E+04 | 4,28E+04 | 4,30E+04 | 4,08E+04 | 4,83E+04 |
| 303 | 0,795 | 1,3 | 9,32E+04 | 8,27E+04 | 1,06E+05 | 9,28E+04 | 1,04E+05 | 1,04E+05 |
| 216 | 0,834 | 1,8 | 8596,424 | 7716,122 | 5651,13 | 9520,579 | 7964,847 | 1,00E+04 |
