# Supplementary material for: Evaluation of the gastrotolerability of ketoprofen, lysine, and gabapentin co-crystal administration in an in vitro model of gastric epithelium: a proteomic update
Source: PLoS One. 2025 Jul 29;20(7):e0328496. doi: 10.1371/journal.pone.0328496 (PMC12306739; doi:10.1371/journal.pone.0328496)
Supplement: S3 Table — Of all the 24 excised spots, 3 spots showed no proteins at all (spots n# 130; 200; 236). (DOCX) [file pone.0328496.s003.docx]

**S3 Table.** **List of identified proteins confidently assigned to 2DE gel spots.** Of all the 24 excised spots, 3 spots showed no proteins at all (spots n# 130; 200; 236).

| Spot | Protein name | Protein accession numbers | Protein molecular weight (Da) | Protein identification probability | Exclusive unique peptide count | Exclusive unique spectrum count | Total spectrum count | % of total spectra | % sequence coverage | Length |
| --- | --- | --- | --- | --- | --- | --- | --- | --- | --- | --- |
| Spot 6 | Involucrin | P07476 | 68.474,60 | 100,00% | 11 | 12 | 16 | 7,55% | 27,90% | 585 |
|  | Isoform 10 of Calpastatin | P20810-10 | 81.002,00 | 100,00% | 4 | 4 | 4 | 1,89% | 7,80% | 754 |
| Spot 25 | Optineurin | Q96CV9 | 65.923,00 | 100,00% | 10 | 11 | 11 | 0,22% | 23,60% | 577 |
|  | Protein kinase C and casein kinase substrate in neurons protein 2 | Q9UNF0 | 55.738,90 | 100,00% | 4 | 5 | 5 | 0,10% | 8,64% | 486 |
|  | Heat shock cognate 71 kDa protein | P11142 | 70.899,80 | 100,00% | 10 | 14 | 52 | 1,03% | 34,50% | 646 |
|  | Heat shock 70 kDa protein 6 | P17066 | 71.028,80 | 100,00% | 1 | 1 | 7 | 0,14% | 9,49% | 643 |
|  | U1 small nuclear ribonucleoprotein 70 kDa | P08621 | 51.558,40 | 100,00% | 5 | 6 | 6 | 0,12% | 11,90% | 437 |
|  | Heat shock 70 kDa protein 1A | P0DMV8 | 70.054,00 | 100,00% | 3 | 3 | 10 | 0,20% | 14,80% | 641 |
|  | Heat shock cognate 71 kDa protein (Fragment) | E9PK54 | 19.955,50 | 99,60% | 1 | 1 | 31 | 0,61% | 72,70% | 183 |
|  | ATP synthase subunit beta, mitochondrial | P06576 | 56.560,60 | 100,00% | 4 | 5 | 5 | 0,10% | 9,07% | 529 |
|  | Src substrate cortactin | Q14247 | 61.585,50 | 100,00% | 3 | 3 | 3 | 0,06% | 5,45% | 550 |
|  | Endoplasmin | P14625 | 92.471,70 | 100,00% | 2 | 2 | 3 | 0,06% | 4,23% | 803 |
|  | Transcriptional coactivator YAP1 | P46937 | 54.461,50 | 100,00% | 3 | 4 | 5 | 0,10% | 10,70% | 504 |
|  | Ezrin OS=Homo sapiens | P15311 | 69.414,70 | 100,00% | 7 | 8 | 10 | 0,20% | 14,00% | 586 |
|  | Heat shock 70 kDa protein 1-like | P34931 | 70.377,10 | 100,00% | 2 | 2 | 13 | 0,26% | 14,80% | 641 |
|  | Heterogeneous nuclear ribonucleoprotein Q | O60506 | 69.603,50 | 100,00% | 5 | 5 | 6 | 0,12% | 8,03% | 623 |
|  | Laminin subunit gamma-2 | Q13753 | 130.976,10 | 100,00% | 3 | 3 | 3 | 0,06% | 3,86% | 1193 |
|  | Stress-70 protein, mitochondrial | P38646 | 73.681,30 | 100,00% | 5 | 5 | 5 | 0,10% | 9,87% | 679 |
|  | Synaptotagmin-like protein 1 | Q8IYJ3 | 61.857,20 | 100,00% | 3 | 4 | 4 | 0,08% | 6,23% | 562 |
|  | Prelamin-A/C | P02545 | 74.140,70 | 100,00% | 22 | 29 | 30 | 0,59% | 38,30% | 664 |
|  | Protein disulfide-isomerase A4 | P13667 | 72.934,00 | 100,00% | 6 | 6 | 7 | 0,14% | 12,70% | 645 |
|  | Endoplasmic reticulum chaperone BiP | P11021 | 72.334,70 | 100,00% | 57 | 95 | 231 | 4,57% | 60,90% | 654 |
|  | Protein Red | Q13123 | 65.605,10 | 100,00% | 3 | 3 | 3 | 0,06% | 7,18% | 557 |
|  | Heterogeneous nuclear ribonucleoprotein M | P52272 | 77.517,30 | 100,00% | 4 | 4 | 4 | 0,08% | 7,95% | 730 |
|  | Peptidyl-prolyl cis-trans isomerase FKBP9 | O95302 | 63.084,80 | 100,00% | 3 | 3 | 3 | 0,06% | 6,14% | 570 |
|  | DBIRD complex subunit ZNF326 | Q5BKZ1 | 65.653,50 | 100,00% | 3 | 3 | 3 | 0,06% | 8,59% | 582 |
| Spot 51 | Nucleobindin-1 | Q02818 | 53.879,60 | 100,00% | 4 | 5 | 5 | 0,11% | 10,40% | 461 |
|  | Non-POU domain-containing octamer-binding protein | Q15233 | 54.231,60 | 100,00% | 6 | 8 | 9 | 0,19% | 17,00% | 471 |
|  | Isoform 2 of Prolyl 4-hydroxylase subunit alpha-1 | P13674-2 | 60.969,30 | 100,00% | 7 | 8 | 8 | 0,17% | 16,50% | 534 |
|  | Tubulin beta chain | P07437 | 49.670,60 | 98,50% | 0 | 0 | 12 | 0,26% | 22,70% | 444 |
|  | Protein kinase C and casein kinase substrate in neurons protein 2 | Q9UNF0 | 55.738,90 | 100,00% | 9 | 10 | 10 | 0,22% | 20,80% | 486 |
|  | Tubulin alpha-4A chain | P68366 | 49.924,60 | 100,00% | 2 | 2 | 6 | 0,13% | 15,80% | 448 |
|  | Ubiquitin carboxyl-terminal hydrolase 14 | P54578 | 56.070,30 | 100,00% | 8 | 8 | 8 | 0,17% | 22,10% | 494 |
|  | Isoform 3 of Heterogeneous nuclear ribonucleoprotein K | P61978-3 | 48.564,60 | 100,00% | 16 | 23 | 44 | 0,95% | 36,40% | 440 |
|  | Prolyl 4-hydroxylase subunit alpha-2 | O15460 | 60.902,90 | 100,00% | 4 | 5 | 5 | 0,11% | 10,30% | 535 |
|  | Heat shock cognate 71 kDa protein | P11142 | 70.899,80 | 100,00% | 2 | 3 | 7 | 0,15% | 10,50% | 646 |
|  | T-complex protein 1 subunit zeta | P40227 | 58.025,30 | 100,00% | 5 | 5 | 5 | 0,11% | 11,90% | 531 |
|  | Heat shock 70 kDa protein 1A | P0DMV8 | 70.054,00 | 99,00% | 0 | 0 | 3 | 0,06% | 5,62% | 641 |
|  | Splicing factor 3A subunit 3 | Q12874 | 58.851,30 | 100,00% | 10 | 14 | 14 | 0,30% | 18,40% | 501 |
|  | Serine/threonine-protein kinase 4 | Q13043 | 55.631,30 | 100,00% | 2 | 2 | 3 | 0,06% | 8,01% | 487 |
|  | Actin, cytoplasmic 2 | P63261 | 41.793,90 | 100,00% | 1 | 1 | 27 | 0,58% | 44,30% | 375 |
|  | ATP synthase subunit beta, mitochondrial | P06576 | 56.560,60 | 100,00% | 5 | 5 | 6 | 0,13% | 12,30% | 529 |
|  | T-complex protein 1 subunit epsilon | P48643 | 59.672,40 | 100,00% | 7 | 9 | 10 | 0,22% | 13,70% | 541 |
|  | Serine/threonine-protein phosphatase 2A 65 kDa regulatory subunit A alpha isoform | P30153 | 65.309,70 | 100,00% | 6 | 8 | 9 | 0,19% | 11,70% | 589 |
|  | Alpha-ketoglutarate-dependent dioxygenase FTO | Q9C0B1 | 58.282,80 | 100,00% | 4 | 4 | 4 | 0,09% | 10,90% | 505 |
|  | Pyruvate kinase PKM | P14618 | 57.937,50 | 100,00% | 20 | 26 | 33 | 0,71% | 39,00% | 531 |
|  | Actin, aortic smooth muscle | P62736 | 42.020,10 | 100,00% | 0 | 0 | 17 | 0,37% | 21,50% | 377 |
|  | T-complex protein 1 subunit theta | P50990 | 59.621,00 | 100,00% | 15 | 18 | 19 | 0,41% | 37,00% | 548 |
|  | NEDD8-activating enzyme E1 regulatory subunit | Q13564 | 60.248,30 | 100,00% | 5 | 5 | 5 | 0,11% | 11,00% | 534 |
|  | Target of Myb protein 1 | O60784 | 53.819,10 | 100,00% | 4 | 4 | 5 | 0,11% | 8,94% | 492 |
|  | 60 kDa heat shock protein, mitochondrial | P10809 | 61.055,70 | 100,00% | 64 | 100 | 197 | 4,23% | 75,40% | 573 |
|  | Tubulin beta-4B chain | P68371 | 49.830,70 | 100,00% | 2 | 2 | 15 | 0,32% | 28,80% | 445 |
|  | Rab GDP dissociation inhibitor alpha | P31150 | 50.584,10 | 100,00% | 2 | 2 | 4 | 0,09% | 10,50% | 447 |
|  | Endoplasmin | P14625 | 92.471,70 | 100,00% | 4 | 4 | 4 | 0,09% | 5,23% | 803 |
|  | Zinc finger CCCH domain-containing protein 15 | Q8WU90 | 48.603,70 | 100,00% | 5 | 6 | 6 | 0,13% | 14,60% | 426 |
|  | Isoform 2 of Tropomyosin alpha-3 chain | P06753-2 | 29.033,30 | 100,00% | 3 | 3 | 4 | 0,09% | 21,40% | 248 |
|  | Eukaryotic translation initiation factor 3 subunit F | O00303 | 37.563,50 | 100,00% | 4 | 4 | 4 | 0,09% | 13,70% | 357 |
|  | TGF-beta-activated kinase 1 and MAP3K7-binding protein 1 | Q15750 | 54.644,00 | 100,00% | 9 | 11 | 14 | 0,30% | 21,60% | 504 |
|  | F-box-like/WD repeat-containing protein TBL1XR1 | Q9BZK7 | 55.594,90 | 100,00% | 11 | 17 | 18 | 0,39% | 26,50% | 514 |
|  | Serine/threonine-protein kinase 3 | Q13188 | 56.302,50 | 100,00% | 4 | 4 | 6 | 0,13% | 11,80% | 491 |
|  | Actin, cytoplasmic 1 | P60709 | 41.737,80 | 100,00% | 1 | 1 | 27 | 0,58% | 44,30% | 375 |
|  | Peptidyl-prolyl cis-trans isomerase FKBP4 | Q02790 | 51.805,90 | 100,00% | 13 | 14 | 17 | 0,37% | 27,90% | 459 |
|  | Endoplasmic reticulum chaperone BiP | P11021 | 72.334,70 | 100,00% | 3 | 3 | 3 | 0,06% | 6,57% | 654 |
|  | Calreticulin | P27797 | 48.142,90 | 100,00% | 9 | 13 | 15 | 0,32% | 24,00% | 417 |
|  | Poly(U)-binding-splicing factor PUF60 | Q9UHX1 | 59.875,60 | 100,00% | 8 | 8 | 10 | 0,22% | 18,10% | 559 |
|  | Nucleosome assembly protein 1-like 4 | Q99733 | 42.823,90 | 100,00% | 5 | 6 | 7 | 0,15% | 15,20% | 375 |
|  | Protein disulfide-isomerase | P07237 | 57.118,10 | 100,00% | 11 | 12 | 12 | 0,26% | 21,90% | 508 |
|  | Serine/arginine-rich splicing factor 6 | Q13247 | 39.588,40 | 100,00% | 2 | 2 | 3 | 0,06% | 9,01% | 344 |
| Spot 60 | Protein disulfide-isomerase A3 | P30101 | 56.784,60 | 100,00% | 8 | 8 | 20 | 17,10% | 21,80% | 505 |
| Spot 66 | T-complex protein 1 subunit beta | P78371 | 57.489,90 | 100,00% | 12 | 14 | 21 | 0,28% | 25,00% | 535 |
|  | RuvB-like 1 | Q9Y265 | 50.229,40 | 100,00% | 3 | 3 | 3 | 0,04% | 7,68% | 456 |
|  | Protein disulfide-isomerase A3 | P30101 | 56.784,60 | 100,00% | 1 | 1 | 3 | 0,04% | 7,13% | 505 |
|  | Aldehyde dehydrogenase X, mitochondrial | P30837 | 57.206,60 | 100,00% | 7 | 8 | 10 | 0,13% | 13,20% | 517 |
|  | Pyruvate dehydrogenase protein X component, mitochondrial | O00330 | 54.123,80 | 100,00% | 6 | 7 | 7 | 0,09% | 13,20% | 501 |
|  | ATP synthase subunit alpha, mitochondrial | P25705 | 59.752,10 | 100,00% | 3 | 3 | 3 | 0,04% | 5,97% | 553 |
|  | Tubulin beta-4B chain | P68371 | 49.830,70 | 99,90% | 1 | 1 | 3 | 0,04% | 7,42% | 445 |
| Spot 86 | Mitochondrial-processing peptidase subunit beta | O75439 | 54.367,10 | 100,00% | 7 | 8 | 10 | 0,15% | 14,90% | 489 |
|  | Arfaptin-1 | P53367 | 41.739,50 | 100,00% | 1 | 1 | 3 | 0,04% | 10,20% | 373 |
|  | Vasodilator-stimulated phosphoprotein | P50552 | 39.829,10 | 100,00% | 6 | 7 | 12 | 0,18% | 25,00% | 380 |
|  | Actin-related protein 3 | P61158 | 47.371,90 | 100,00% | 3 | 3 | 3 | 0,04% | 9,33% | 418 |
|  | Alpha-enolase | P06733 | 47.170,20 | 100,00% | 10 | 11 | 15 | 0,22% | 26,00% | 434 |
|  | Aldehyde dehydrogenase X, mitochondrial | P30837 | 57.206,60 | 100,00% | 3 | 3 | 3 | 0,04% | 6,58% | 517 |
|  | Non-specific lipid-transfer protein | P22307 | 58.994,60 | 100,00% | 4 | 5 | 7 | 0,10% | 6,22% | 547 |
|  | ATP synthase subunit alpha, mitochondrial | P25705 | 59.752,10 | 100,00% | 4 | 4 | 4 | 0,06% | 8,14% | 553 |
|  | Proliferation-associated protein 2G4 | Q9UQ80 | 43.786,70 | 100,00% | 7 | 8 | 11 | 0,16% | 19,80% | 394 |
| Spot 93 | Synaptic vesicle membrane protein VAT-1 homolog | Q99536 | 41.919,80 | 100,00% | 3 | 3 | 3 | 0,04% | 10,40% | 393 |
|  | DnaJ homolog subfamily A member 1 | P31689 | 44.868,60 | 100,00% | 4 | 5 | 6 | 0,09% | 12,30% | 397 |
|  | Fumarate hydratase, mitochondrial | P07954 | 54.637,80 | 100,00% | 9 | 11 | 12 | 0,17% | 19,20% | 510 |
|  | Alpha-enolase | P06733 | 47.170,20 | 100,00% | 37 | 65 | 134 | 1,92% | 70,50% | 434 |
|  | Regulator of chromosome condensation | P18754 | 44.967,80 | 100,00% | 3 | 3 | 3 | 0,04% | 9,50% | 421 |
|  | Tubulin beta-4B chain | P68371 | 49.830,70 | 100,00% | 2 | 2 | 6 | 0,09% | 13,30% | 445 |
|  | Glyceraldehyde-3-phosphate dehydrogenase | P04406 | 36.053,40 | 100,00% | 3 | 3 | 4 | 0,06% | 13,70% | 335 |
|  | Protein disulfide-isomerase A6 | Q15084 | 48.122,30 | 100,00% | 5 | 6 | 6 | 0,09% | 13,20% | 440 |
|  | Actin-related protein 3 | P61158 | 47.371,90 | 100,00% | 5 | 5 | 5 | 0,07% | 13,90% | 418 |
|  | Calcium/calmodulin-dependent protein kinase type II subunit delta | Q13557 | 56.370,70 | 100,00% | 4 | 4 | 4 | 0,06% | 8,22% | 499 |
|  | SH3 domain-containing kinase-binding protein 1 (Fragment) | Q5JPT2 | 73.128,90 | 100,00% | 15 | 18 | 26 | 0,37% | 26,50% | 665 |
|  | Histone-binding protein RBBP7 | Q16576 | 47.820,00 | 100,00% | 2 | 2 | 3 | 0,04% | 8,94% | 425 |
|  | Phosphoglycerate kinase 1 | P00558 | 44.615,30 | 100,00% | 4 | 4 | 4 | 0,06% | 12,70% | 417 |
|  | Thyroid receptor-interacting protein 6 | Q15654 | 50.286,70 | 100,00% | 3 | 4 | 4 | 0,06% | 9,03% | 476 |
|  | Trifunctional enzyme subunit beta, mitochondrial | P55084 | 51.296,00 | 100,00% | 13 | 16 | 19 | 0,27% | 27,40% | 474 |
|  | Tubulin alpha-4A chain | P68366 | 49.924,60 | 100,00% | 1 | 1 | 3 | 0,04% | 8,26% | 448 |
|  | Elongation factor 1-alpha 1 | P68104 | 50.470,60 | 100,00% | 3 | 3 | 4 | 0,06% | 6,06% | 463 |
|  | Ladinin-1 | O00515 | 57.131,80 | 100,00% | 3 | 3 | 3 | 0,04% | 10,30% | 517 |
|  | Plasminogen activator inhibitor 1 RNA-binding protein | Q8NC51 | 44.965,80 | 100,00% | 3 | 3 | 3 | 0,04% | 10,00% | 408 |
|  | Histone-binding protein RBBP4 | Q09028 | 47.655,80 | 100,00% | 2 | 2 | 3 | 0,04% | 9,18% | 425 |
|  | S-adenosylmethionine synthase isoform type-2 | P31153 | 43.660,90 | 100,00% | 4 | 4 | 4 | 0,06% | 11,90% | 395 |
|  | Proliferation-associated protein 2G4 | Q9UQ80 | 43.786,70 | 100,00% | 5 | 6 | 6 | 0,09% | 15,20% | 394 |
|  | Serine/arginine-rich splicing factor 1 | J3KTL2 | 27.745,10 | 100,00% | 3 | 3 | 4 | 0,06% | 13,30% | 248 |
|  | Endophilin-B2 | Q9NR46 | 43.975,20 | 100,00% | 3 | 3 | 3 | 0,04% | 9,62% | 395 |
|  | Prelamin-A/C | P02545 | 74.140,70 | 100,00% | 5 | 5 | 5 | 0,07% | 10,10% | 664 |
|  | Serine hydroxymethyltransferase, mitochondrial | P34897 | 55.994,70 | 100,00% | 5 | 5 | 6 | 0,09% | 11,90% | 504 |
|  | Mitochondrial-processing peptidase subunit beta | O75439 | 54.367,10 | 100,00% | 3 | 3 | 3 | 0,04% | 7,98% | 489 |
|  | RuvB-like 2 | Q9Y230 | 51.158,10 | 100,00% | 3 | 3 | 3 | 0,04% | 7,99% | 463 |
|  | Suprabasin | Q6UWP8 | 60.537,20 | 100,00% | 3 | 5 | 7 | 0,10% | 10,80% | 590 |
|  | Lupus La protein | P05455 | 46.839,00 | 100,00% | 3 | 3 | 3 | 0,04% | 8,82% | 408 |
|  | Vasodilator-stimulated phosphoprotein | P50552 | 39.829,10 | 100,00% | 21 | 35 | 56 | 0,80% | 56,30% | 380 |
|  | Glutamate dehydrogenase 1, mitochondrial | P00367 | 61.399,70 | 100,00% | 14 | 16 | 18 | 0,26% | 26,70% | 558 |
|  | ATP synthase subunit alpha, mitochondrial | P25705 | 59.752,10 | 100,00% | 18 | 25 | 28 | 0,40% | 33,10% | 553 |
|  | Annexin A11 | P50995 | 54.391,70 | 100,00% | 4 | 4 | 4 | 0,06% | 8,71% | 505 |
|  | Flotillin-1 | O75955 | 47.354,80 | 100,00% | 3 | 3 | 3 | 0,04% | 9,13% | 427 |
|  | Heterogeneous nuclear ribonucleoprotein D0 | Q14103 | 38.434,50 | 100,00% | 6 | 6 | 8 | 0,11% | 16,90% | 355 |
|  | Rab GDP dissociation inhibitor beta | P50395 | 50.665,50 | 100,00% | 6 | 6 | 8 | 0,11% | 19,30% | 445 |
|  | Egl nine homolog 1 | Q9GZT9 | 46.021,50 | 100,00% | 3 | 3 | 5 | 0,07% | 10,60% | 426 |
|  | Septin-7 | Q16181 | 50.680,30 | 100,00% | 6 | 7 | 7 | 0,10% | 16,70% | 437 |
|  | Gamma-enolase | P09104 | 47.269,70 | 100,00% | 6 | 7 | 13 | 0,19% | 21,00% | 434 |
|  | Pre-mRNA-splicing factor RBM22 | Q9NW64 | 46.896,60 | 100,00% | 3 | 3 | 3 | 0,04% | 9,05% | 420 |
|  | Fascin | Q16658 | 54.530,30 | 100,00% | 4 | 4 | 4 | 0,06% | 10,10% | 493 |
|  | ATP synthase subunit beta, mitochondrial | P06576 | 56.560,60 | 100,00% | 11 | 16 | 21 | 0,30% | 29,10% | 529 |
|  | Dihydrolipoyllysine-residue succinyltransferase component of 2-oxoglutarate dehydrogenase complex, mitochondrial | P36957 | 48.755,20 | 100,00% | 3 | 3 | 3 | 0,04% | 6,40% | 453 |
|  | Fructose-bisphosphate aldolase A | P04075 | 39.420,60 | 100,00% | 5 | 5 | 5 | 0,07% | 14,30% | 364 |
|  | Pyruvate kinase PKM | P14618 | 57.937,50 | 100,00% | 5 | 6 | 6 | 0,09% | 10,20% | 531 |
|  | Coronin-1C | Q9ULV4 | 53.250,30 | 100,00% | 9 | 11 | 12 | 0,17% | 18,60% | 474 |
|  | NADPH:adrenodoxin oxidoreductase, mitochondrial | P22570 | 53.836,50 | 100,00% | 6 | 6 | 7 | 0,10% | 15,90% | 491 |
| Spot 115 | Fructose-bisphosphate aldolase C | P09972 | 39.456,20 | 100,00% | 3 | 3 | 3 | 1,21% | 7,42% | 364 |
|  | Fructose-bisphosphate aldolase A | P04075 | 39.420,60 | 100,00% | 3 | 3 | 4 | 1,61% | 7,42% | 364 |
|  | Isoform 2 of Heterogeneous nuclear ribonucleoprotein A/B | Q99729-2 | 30.303,00 | 100,00% | 2 | 2 | 3 | 1,21% | 10,80% | 280 |
|  | LIM and SH3 domain protein 1 | Q14847 | 29.716,80 | 100,00% | 3 | 3 | 4 | 1,61% | 14,90% | 261 |
| Spot 126 | Quinone oxidoreductase | Q08257 | 35.207,30 | 100,00% | 5 | 7 | 8 | 0,18% | 14,00% | 329 |
|  | Heterogeneous nuclear ribonucleoproteins A2/B1 | P22626 | 37.430,30 | 100,00% | 7 | 8 | 11 | 0,25% | 24,60% | 353 |
|  | Serine/threonine-protein phosphatase PP1-alpha catalytic subunit | P62136 | 37.513,90 | 100,00% | 1 | 2 | 6 | 0,13% | 8,79% | 330 |
|  | AH receptor-interacting protein | O00170 | 37.636,40 | 100,00% | 7 | 10 | 10 | 0,22% | 20,90% | 330 |
|  | L-lactate dehydrogenase A chain | P00338 | 36.689,20 | 100,00% | 3 | 3 | 3 | 0,07% | 10,20% | 332 |
|  | Heterogeneous nuclear ribonucleoprotein H3 | P31942 | 36.927,60 | 100,00% | 3 | 3 | 3 | 0,07% | 10,10% | 346 |
|  | Isoform 2 of 3-mercaptopyruvate sulfurtransferase | P25325-2 | 35.250,00 | 100,00% | 4 | 6 | 7 | 0,16% | 14,50% | 317 |
|  | Glyoxylate reductase/hydroxypyruvate reductase | Q9UBQ7 | 35.669,10 | 100,00% | 4 | 4 | 5 | 0,11% | 14,30% | 328 |
|  | PDZ and LIM domain protein 1 | O00151 | 36.071,30 | 100,00% | 8 | 10 | 10 | 0,22% | 26,40% | 329 |
|  | Protein CDV3 homolog | Q9UKY7 | 27.334,70 | 100,00% | 5 | 6 | 6 | 0,13% | 34,90% | 258 |
|  | Pyruvate kinase PKM | P14618 | 57.937,50 | 100,00% | 3 | 4 | 4 | 0,09% | 6,59% | 531 |
|  | Galactokinase | P51570 | 42.272,10 | 100,00% | 4 | 4 | 4 | 0,09% | 11,70% | 392 |
|  | Calponin-3 | Q15417 | 36.414,60 | 100,00% | 3 | 3 | 3 | 0,07% | 13,40% | 329 |
|  | UDP-glucose 4-epimerase | Q14376 | 38.282,00 | 100,00% | 6 | 7 | 8 | 0,18% | 21,30% | 348 |
|  | Malate dehydrogenase, mitochondrial | P40926 | 35.503,70 | 100,00% | 9 | 11 | 13 | 0,29% | 27,80% | 338 |
|  | Annexin A2 | P07355 | 38.606,10 | 100,00% | 17 | 21 | 23 | 0,52% | 46,30% | 339 |
|  | Charged multivesicular body protein 4c | Q96CF2 | 26.411,60 | 100,00% | 3 | 4 | 6 | 0,13% | 20,60% | 233 |
|  | Aldo-keto reductase family 1 member A1 | P14550 | 36.573,60 | 100,00% | 4 | 4 | 6 | 0,13% | 13,20% | 325 |
|  | Glyceraldehyde-3-phosphate dehydrogenase | P04406 | 36.053,40 | 100,00% | 11 | 16 | 24 | 0,54% | 33,70% | 335 |
|  | m7GpppX diphosphatase | Q96C86 | 38.609,80 | 100,00% | 3 | 4 | 4 | 0,09% | 13,90% | 337 |
|  | LIM and SH3 domain protein 1 | Q14847 | 29.716,80 | 100,00% | 22 | 32 | 63 | 1,41% | 57,10% | 261 |
|  | Annexin A1 | P04083 | 38.715,90 | 100,00% | 27 | 36 | 55 | 1,23% | 63,60% | 346 |
|  | Quinone oxidoreductase PIG3 | Q53FA7 | 35.537,30 | 100,00% | 4 | 4 | 4 | 0,09% | 14,80% | 332 |
|  | Phosphotriesterase-related protein | Q96BW5 | 39.018,90 | 100,00% | 4 | 4 | 4 | 0,09% | 13,50% | 349 |
|  | Crk-like protein | P46109 | 33.777,30 | 100,00% | 6 | 7 | 10 | 0,22% | 26,40% | 303 |
|  | Fructose-bisphosphate aldolase A | P04075 | 39.420,60 | 100,00% | 5 | 6 | 7 | 0,16% | 10,40% | 364 |
|  | Transaldolase | P37837 | 37.541,70 | 100,00% | 13 | 17 | 18 | 0,40% | 36,80% | 337 |
|  | F-actin-capping protein subunit alpha-2 | P47755 | 32.949,20 | 100,00% | 2 | 2 | 3 | 0,07% | 14,00% | 286 |
|  | 3-hydroxyisobutyryl-CoA hydrolase, mitochondrial | Q6NVY1 | 43.484,00 | 100,00% | 3 | 4 | 4 | 0,09% | 9,07% | 386 |
|  | Elongation factor 1-delta | P29692 | 31.121,90 | 100,00% | 4 | 4 | 4 | 0,09% | 21,00% | 281 |
|  | Isocitrate dehydrogenase [NAD] subunit alpha, mitochondrial | P50213 | 39.592,50 | 100,00% | 3 | 3 | 3 | 0,07% | 8,74% | 366 |
|  | Isoform 2 of Nebulette | O76041-2 | 31.195,40 | 98,90% | 1 | 1 | 4 | 0,09% | 10,40% | 270 |
|  | Oxygen-dependent coproporphyrinogen-III oxidase, mitochondrial | P36551 | 50.152,00 | 100,00% | 3 | 3 | 3 | 0,07% | 8,15% | 454 |
|  | Poly(rC)-binding protein 2 | F8VZX2 | 38.580,90 | 99,90% | 1 | 1 | 4 | 0,09% | 12,30% | 365 |
|  | Phosphoglycerate kinase 1 | P00558 | 44.615,30 | 100,00% | 8 | 10 | 10 | 0,22% | 22,80% | 417 |
|  | Adaptin ear-binding coat-associated protein 1 | Q8NC96 | 29.737,90 | 100,00% | 5 | 6 | 7 | 0,16% | 16,40% | 275 |
|  | F-actin-capping protein subunit alpha-1 | P52907 | 32.923,20 | 100,00% | 2 | 2 | 3 | 0,07% | 14,00% | 286 |
|  | Alpha-enolase | P06733 | 47.170,20 | 100,00% | 6 | 7 | 8 | 0,18% | 18,20% | 434 |
|  | PDZ domain-containing protein GIPC1 | O14908 | 36.050,60 | 100,00% | 3 | 3 | 3 | 0,07% | 11,70% | 333 |
|  | Aminoacyl tRNA synthase complex-interacting multifunctional protein 1 | Q12904 | 34.353,00 | 100,00% | 3 | 4 | 4 | 0,09% | 11,90% | 312 |
|  | RNA-binding protein Raly (Fragment) | Q5QPL9 | 32.463,90 | 100,00% | 6 | 7 | 7 | 0,16% | 24,90% | 306 |
|  | Serine/arginine-rich splicing factor 1 | J3KTL2 | 27.745,10 | 100,00% | 3 | 3 | 3 | 0,07% | 12,10% | 248 |
|  | Isoform 2 of Heterogeneous nuclear ribonucleoprotein D-like | O14979-2 | 33.589,60 | 100,00% | 6 | 7 | 8 | 0,18% | 29,20% | 301 |
|  | Serine/threonine-protein phosphatase PP1-beta catalytic subunit | P62140 | 37.188,30 | 100,00% | 2 | 2 | 6 | 0,13% | 13,50% | 327 |
| Spot 130 | **NO ASSIGNEMENT** | | | | | | | | | |
| Spot 155 | 14-3-3 protein gamma | P61981 | 28.303,10 | 100,00% | 3 | 3 | 3 | 0,10% | 13,80% | 247 |
|  | Heat shock protein beta-1 | P04792 | 22.782,60 | 100,00% | 15 | 20 | 47 | 1,49% | 68,30% | 205 |
|  | Cathepsin D | P07339 | 44.553,00 | 100,00% | 3 | 3 | 3 | 0,10% | 8,98% | 412 |
|  | Peroxiredoxin-6 | P30041 | 25.036,10 | 100,00% | 5 | 5 | 6 | 0,19% | 27,20% | 224 |
|  | 6-phosphogluconolactonase | O95336 | 27.547,50 | 100,00% | 3 | 3 | 5 | 0,16% | 16,30% | 258 |
|  | 14-3-3 protein zeta/delta | P63104 | 27.745,90 | 100,00% | 4 | 4 | 4 | 0,13% | 21,20% | 245 |
|  | Triosephosphate isomerase | P60174 | 30.790,80 | 100,00% | 3 | 3 | 3 | 0,10% | 12,20% | 286 |
|  | Enoyl-CoA hydratase, mitochondrial | P30084 | 31.388,20 | 100,00% | 4 | 4 | 5 | 0,16% | 12,40% | 290 |
| Spot 178 | 40S ribosomal protein S13 | P62277 | 17.223,30 | 100,00% | 3 | 4 | 4 | 0,20% | 15,20% | 151 |
|  | 40S ribosomal protein S18 | P62269 | 17.719,30 | 100,00% | 6 | 7 | 8 | 0,40% | 32,90% | 152 |
|  | Nucleoside diphosphate kinase A | P15531 | 17.148,80 | 100,00% | 6 | 9 | 14 | 0,69% | 46,70% | 152 |
|  | Isoform 2 of Tropomyosin alpha-3 chain | P06753-2 | 29.033,30 | 100,00% | 3 | 3 | 3 | 0,15% | 14,90% | 248 |
|  | 60S ribosomal protein L12 | P30050 | 17.819,10 | 100,00% | 3 | 3 | 5 | 0,25% | 24,20% | 165 |
|  | Apoptosis-associated speck-like protein containing a CARD | Q9ULZ3 | 21.627,80 | 100,00% | 3 | 3 | 3 | 0,15% | 12,30% | 195 |
|  | Stathmin | P16949 | 17.303,00 | 100,00% | 3 | 3 | 3 | 0,15% | 14,80% | 149 |
|  | 40S ribosomal protein S11 | P62280 | 18.431,30 | 100,00% | 3 | 3 | 3 | 0,15% | 18,40% | 158 |
|  | Cofilin-1 | P23528 | 18.503,20 | 100,00% | 4 | 4 | 5 | 0,25% | 27,10% | 166 |
|  | Superoxide dismutase [Cu-Zn] | P00441 | 15.935,30 | 100,00% | 7 | 9 | 17 | 0,84% | 55,80% | 154 |
| Spot 200 | **NO ASSIGNEMENT** | | | | | | | | | |
| Spot 216 | Glycerol-3-phosphate dehydrogenase, mitochondrial | P43304 | 80.854,60 | 100,00% | 3 | 3 | 4 | 0,12% | 5,23% | 727 |
|  | Ladinin-1 | O00515 | 57.131,80 | 100,00% | 3 | 3 | 3 | 0,09% | 5,03% | 517 |
|  | Tripartite motif-containing protein 29 | Q14134 | 65.835,70 | 100,00% | 6 | 6 | 6 | 0,18% | 11,10% | 588 |
|  | Coiled-coil domain-containing protein 6 | Q16204 | 53.292,00 | 100,00% | 5 | 5 | 6 | 0,18% | 11,00% | 474 |
|  | Stress-induced-phosphoprotein 1 | P31948 | 62.642,10 | 100,00% | 47 | 66 | 105 | 3,07% | 65,60% | 543 |
|  | Cleavage stimulation factor subunit 2 | P33240 | 60.959,10 | 100,00% | 4 | 4 | 5 | 0,15% | 7,97% | 577 |
|  | Prelamin-A/C | P02545 | 74.140,70 | 100,00% | 24 | 32 | 35 | 1,02% | 37,00% | 664 |
|  | Paraspeckle component 1 | Q8WXF1 | 58.744,50 | 100,00% | 3 | 3 | 4 | 0,12% | 6,50% | 523 |
|  | Bifunctional purine biosynthesis protein ATIC | P31939 | 64.616,50 | 100,00% | 13 | 14 | 20 | 0,59% | 29,20% | 592 |
|  | WD repeat-containing protein 1 | O75083 | 66.193,20 | 100,00% | 7 | 7 | 8 | 0,23% | 15,30% | 606 |
| Spot 223 | Isoform 3 of Calcyphosin | Q13938-4 | 20.967,70 | 100,00% | 5 | 5 | 17 | 11,90% | 32,80% | 189 |
| Spot 228 | Septin-11 | Q9NVA2 | 49.399,10 | 100,00% | 3 | 4 | 5 | 0,08% | 7,69% | 429 |
|  | Prelamin-A/C | P02545 | 74.140,70 | 100,00% | 3 | 3 | 3 | 0,05% | 7,08% | 664 |
|  | Alpha-enolase | P06733 | 47.170,20 | 100,00% | 22 | 29 | 53 | 0,84% | 52,50% | 434 |
| Spot 236 | **NO ASSIGNEMENT** | | | | | | | | | |
| Spot 241 | 3-hydroxyacyl-CoA dehydrogenase type-2 | Q99714 | 26.923,10 | 100,00% | 4 | 4 | 4 | 2,41% | 20,30% | 261 |
|  | Triosephosphate isomerase | P60174 | 30.790,80 | 100,00% | 14 | 19 | 46 | 27,70% | 54,20% | 286 |
| Spot 244 | Methylmalonate-semialdehyde dehydrogenase [acylating], mitochondrial | Q02252 | 57.840,30 | 100,00% | 5 | 5 | 6 | 0,12% | 11,40% | 535 |
|  | Aldehyde dehydrogenase X, mitochondrial | P30837 | 57.206,60 | 100,00% | 5 | 5 | 6 | 0,12% | 13,90% | 517 |
|  | Aldehyde dehydrogenase, dimeric NADP-preferring | P30838 | 50.395,70 | 100,00% | 8 | 10 | 12 | 0,24% | 24,50% | 453 |
|  | Annexin A2 | P07355 | 38.606,10 | 100,00% | 4 | 4 | 5 | 0,10% | 11,20% | 339 |
|  | Protein disulfide-isomerase A3 | P30101 | 56.784,60 | 100,00% | 18 | 24 | 43 | 0,85% | 42,20% | 505 |
|  | Alpha-enolase | P06733 | 47.170,20 | 100,00% | 8 | 10 | 12 | 0,24% | 28,30% | 434 |
|  | Vasodilator-stimulated phosphoprotein | P50552 | 39.829,10 | 100,00% | 10 | 11 | 12 | 0,24% | 36,80% | 380 |
|  | Heterogeneous nuclear ribonucleoprotein H2 | P55795 | 49.264,10 | 100,00% | 3 | 4 | 9 | 0,18% | 22,00% | 449 |
|  | Isoform A of Serine/threonine-protein kinase 24 | Q9Y6E0-2 | 45.838,10 | 100,00% | 4 | 4 | 6 | 0,12% | 17,40% | 412 |
|  | 60 kDa heat shock protein, mitochondrial | P10809 | 61.055,70 | 100,00% | 4 | 5 | 5 | 0,10% | 11,00% | 573 |
|  | Tubulin beta-4B chain | P68371 | 49.830,70 | 100,00% | 3 | 4 | 50 | 0,99% | 36,20% | 445 |
|  | Cytoplasmic dynein 1 light intermediate chain 2 | O43237 | 54.099,80 | 100,00% | 3 | 3 | 3 | 0,06% | 8,13% | 492 |
|  | Alpha-aminoadipic semialdehyde dehydrogenase | P49419 | 58.487,60 | 100,00% | 9 | 9 | 10 | 0,20% | 18,70% | 539 |
|  | Glyceraldehyde-3-phosphate dehydrogenase | P04406 | 36.053,40 | 100,00% | 4 | 5 | 5 | 0,10% | 17,30% | 335 |
|  | Inosine-5'-monophosphate dehydrogenase 2 | P12268 | 55.806,50 | 100,00% | 9 | 12 | 12 | 0,24% | 19,30% | 514 |
|  | UTP--glucose-1-phosphate uridylyltransferase | Q16851 | 56.942,00 | 100,00% | 11 | 13 | 16 | 0,32% | 27,20% | 508 |
|  | Actin-related protein 3 | P61158 | 47.371,90 | 100,00% | 3 | 3 | 3 | 0,06% | 8,37% | 418 |
|  | Actin, cytoplasmic 2 | P63261 | 41.793,90 | 100,00% | 1 | 1 | 18 | 0,36% | 34,70% | 375 |
|  | Aldehyde dehydrogenase, mitochondrial | P05091 | 56.381,40 | 100,00% | 8 | 9 | 10 | 0,20% | 21,30% | 517 |
|  | Protein kinase C and casein kinase substrate in neurons protein 3 | Q9UKS6 | 48.486,70 | 100,00% | 4 | 4 | 4 | 0,08% | 12,70% | 424 |
|  | Phosphoglycerate kinase 1 | P00558 | 44.615,30 | 100,00% | 4 | 4 | 4 | 0,08% | 10,30% | 417 |
|  | Adenylyl cyclase-associated protein 1 | Q01518 | 51.901,60 | 100,00% | 8 | 9 | 9 | 0,18% | 21,10% | 475 |
|  | Isoform 3 of Heterogeneous nuclear ribonucleoprotein K | P61978-3 | 48.564,60 | 100,00% | 7 | 7 | 9 | 0,18% | 21,60% | 440 |
|  | Protein disulfide-isomerase A3 (Fragment) | H7BZJ3 | 13.519,70 | 99,50% | 1 | 1 | 13 | 0,26% | 52,80% | 123 |
|  | Pre-mRNA-processing factor 19 | Q9UMS4 | 55.181,30 | 100,00% | 6 | 7 | 7 | 0,14% | 15,10% | 504 |
|  | Septin-8 | Q92599 | 55.756,40 | 100,00% | 3 | 3 | 4 | 0,08% | 10,10% | 483 |
|  | Vacuolar protein sorting-associated protein 4A | Q9UN37 | 48.899,30 | 100,00% | 4 | 4 | 7 | 0,14% | 17,60% | 437 |
|  | Tubulin alpha-4A chain | P68366 | 49.924,60 | 100,00% | 4 | 4 | 16 | 0,32% | 28,60% | 448 |
|  | Tubulin alpha-1B chain | P68363 | 50.135,70 | 99,80% | 1 | 1 | 20 | 0,40% | 37,50% | 451 |
|  | Tubulin beta-6 chain | Q9BUF5 | 49.857,20 | 100,00% | 5 | 5 | 31 | 0,62% | 30,50% | 446 |
|  | Aspartate--tRNA ligase, cytoplasmic | P14868 | 57.136,70 | 100,00% | 11 | 12 | 14 | 0,28% | 29,70% | 501 |
|  | T-complex protein 1 subunit beta | P78371 | 57.489,90 | 100,00% | 11 | 15 | 19 | 0,38% | 25,40% | 535 |
|  | Septin-11 | Q9NVA2 | 49.399,10 | 100,00% | 6 | 7 | 8 | 0,16% | 14,90% | 429 |
|  | Methanethiol oxidase | Q13228 | 52.392,00 | 100,00% | 5 | 5 | 5 | 0,10% | 12,50% | 472 |
|  | Tubulin beta-3 chain | Q13509 | 50.432,70 | 100,00% | 2 | 2 | 43 | 0,85% | 33,80% | 450 |
|  | RuvB-like 1 | Q9Y265 | 50.229,40 | 100,00% | 9 | 9 | 9 | 0,18% | 21,30% | 456 |
|  | Septin-10 | Q9P0V9 | 52.593,50 | 100,00% | 4 | 4 | 4 | 0,08% | 10,40% | 454 |
|  | Cytosol aminopeptidase | P28838 | 56.167,80 | 100,00% | 8 | 9 | 10 | 0,20% | 19,30% | 519 |
|  | Tubulin beta-2A chain | Q13885 | 49.907,10 | 100,00% | 1 | 1 | 43 | 0,85% | 30,80% | 445 |
|  | Tubulin alpha-1C chain | Q9BQE3 | 49.895,50 | 100,00% | 2 | 2 | 20 | 0,40% | 37,20% | 449 |
|  | Heterogeneous nuclear ribonucleoprotein H | P31943 | 49.229,80 | 100,00% | 5 | 5 | 10 | 0,20% | 31,00% | 449 |
|  | D-3-phosphoglycerate dehydrogenase | O43175 | 56.650,60 | 100,00% | 8 | 8 | 8 | 0,16% | 13,90% | 533 |
|  | Endophilin-B2 | Q9NR46 | 43.975,20 | 100,00% | 5 | 5 | 6 | 0,12% | 15,70% | 395 |
|  | Serine hydroxymethyltransferase, mitochondrial | P34897 | 55.994,70 | 100,00% | 10 | 10 | 12 | 0,24% | 21,40% | 504 |
|  | T-complex protein 1 subunit theta | P50990 | 59.621,00 | 100,00% | 3 | 3 | 3 | 0,06% | 7,12% | 548 |
|  | Actin, cytoplasmic 1 | P60709 | 41.737,80 | 100,00% | 1 | 1 | 18 | 0,36% | 34,70% | 375 |
|  | RuvB-like 2 | Q9Y230 | 51.158,10 | 100,00% | 3 | 3 | 3 | 0,06% | 7,78% | 463 |
|  | 4-trimethylaminobutyraldehyde dehydrogenase | P49189 | 53.802,00 | 100,00% | 6 | 6 | 6 | 0,12% | 18,40% | 494 |
|  | Dipeptidyl peptidase 2 | Q9UHL4 | 54.342,90 | 100,00% | 4 | 4 | 4 | 0,08% | 11,20% | 492 |
|  | Glucose-6-phosphate isomerase | P06744 | 63.148,50 | 100,00% | 8 | 9 | 9 | 0,18% | 18,50% | 558 |
|  | Glutamate dehydrogenase 1, mitochondrial | P00367 | 61.399,70 | 100,00% | 19 | 24 | 26 | 0,52% | 39,60% | 558 |
|  | ATP synthase subunit alpha, mitochondrial | P25705 | 59.752,10 | 100,00% | 21 | 27 | 32 | 0,64% | 41,00% | 553 |
|  | Retinal dehydrogenase 1 | P00352 | 54.862,80 | 100,00% | 11 | 13 | 17 | 0,34% | 24,80% | 501 |
|  | Polypyrimidine tract-binding protein 1 | P26599 | 57.222,50 | 100,00% | 4 | 5 | 5 | 0,10% | 9,79% | 531 |
|  | Actin, aortic smooth muscle | P62736 | 42.020,10 | 99,80% | 0 | 0 | 11 | 0,22% | 19,10% | 377 |
|  | Annexin A11 | P50995 | 54.391,70 | 100,00% | 13 | 16 | 16 | 0,32% | 28,70% | 505 |
|  | Lamina-associated polypeptide 2, isoform alpha | P42166 | 75.493,80 | 100,00% | 3 | 3 | 3 | 0,06% | 5,91% | 694 |
|  | Cytosolic non-specific dipeptidase | Q96KP4 | 52.879,70 | 100,00% | 5 | 5 | 5 | 0,10% | 12,80% | 475 |
|  | Xaa-Pro dipeptidase | P12955 | 54.547,70 | 100,00% | 4 | 4 | 5 | 0,10% | 11,00% | 493 |
|  | Dihydrolipoyl dehydrogenase, mitochondrial | P09622 | 54.177,50 | 100,00% | 6 | 6 | 6 | 0,12% | 14,90% | 509 |
|  | Glutathione synthetase | P48637 | 52.385,90 | 100,00% | 4 | 4 | 4 | 0,08% | 9,92% | 474 |
|  | Eukaryotic translation initiation factor 2 subunit 3 | P41091 | 51.110,20 | 100,00% | 4 | 4 | 4 | 0,08% | 10,40% | 472 |
|  | Peptidyl-prolyl cis-trans isomerase FKBP4 | Q02790 | 51.805,90 | 100,00% | 5 | 5 | 6 | 0,12% | 13,50% | 459 |
|  | Glutathione reductase, mitochondrial | P00390 | 56.257,40 | 100,00% | 6 | 6 | 6 | 0,12% | 15,30% | 522 |
|  | CUGBP Elav-like family member 1 | Q92879 | 52.064,10 | 100,00% | 3 | 3 | 4 | 0,08% | 8,02% | 486 |
|  | Prenylcysteine oxidase 1 | Q9UHG3 | 56.642,20 | 100,00% | 5 | 5 | 5 | 0,10% | 10,70% | 505 |
|  | Fascin | Q16658 | 54.530,30 | 100,00% | 12 | 15 | 16 | 0,32% | 29,20% | 493 |
|  | Tubulin beta chain | P07437 | 49.670,60 | 100,00% | 3 | 3 | 48 | 0,95% | 36,30% | 444 |
|  | V-type proton ATPase subunit B, brain isoform | P21281 | 56.502,10 | 100,00% | 3 | 3 | 4 | 0,08% | 7,44% | 511 |
|  | ATP synthase subunit beta, mitochondrial | P06576 | 56.560,60 | 100,00% | 9 | 9 | 12 | 0,24% | 20,40% | 529 |
|  | Glucose-6-phosphate 1-dehydrogenase | P11413 | 59.258,10 | 100,00% | 5 | 6 | 6 | 0,12% | 10,70% | 515 |
|  | Dihydrolipoyllysine-residue succinyltransferase component of 2-oxoglutarate dehydrogenase complex, mitochondrial | P36957 | 48.755,20 | 100,00% | 5 | 5 | 6 | 0,12% | 11,90% | 453 |
|  | Fructose-bisphosphate aldolase A | P04075 | 39.420,60 | 100,00% | 4 | 5 | 5 | 0,10% | 10,40% | 364 |
|  | Nicotinate phosphoribosyltransferase | Q6XQN6 | 57.578,50 | 100,00% | 5 | 5 | 6 | 0,12% | 11,30% | 538 |
|  | RNA-splicing ligase RtcB homolog | Q9Y3I0 | 55.210,20 | 100,00% | 12 | 13 | 14 | 0,28% | 27,90% | 505 |
|  | Pyruvate kinase PKM | P14618 | 57.937,50 | 100,00% | 17 | 18 | 22 | 0,44% | 33,70% | 531 |
|  | Abl interactor 1 | Q8IZP0 | 55.081,80 | 100,00% | 3 | 3 | 3 | 0,06% | 9,25% | 508 |
|  | Coronin-1C | Q9ULV4 | 53.250,30 | 100,00% | 10 | 12 | 13 | 0,26% | 18,10% | 474 |
|  | Serine--tRNA ligase, mitochondrial | Q9NP81 | 58.283,50 | 100,00% | 3 | 3 | 3 | 0,06% | 8,69% | 518 |
| Spot 263 | Heterogeneous nuclear ribonucleoproteins A2/B1 | P22626 | 37.430,30 | 100,00% | 10 | 11 | 12 | 0,24% | 31,40% | 353 |
|  | Isoform 2 of Nebulette | O76041-2 | 31.195,40 | 99,30% | 1 | 1 | 5 | 0,10% | 10,40% | 270 |
|  | 39S ribosomal protein L39, mitochondrial | Q9NYK5 | 38.712,40 | 100,00% | 11 | 12 | 14 | 0,29% | 33,70% | 338 |
|  | Heterogeneous nuclear ribonucleoprotein H3 | P31942 | 36.927,60 | 100,00% | 19 | 26 | 44 | 0,90% | 60,10% | 346 |
|  | PDZ and LIM domain protein 1 | O00151 | 36.071,30 | 100,00% | 31 | 48 | 100 | 2,03% | 76,60% | 329 |
|  | Methionine adenosyltransferase 2 subunit beta | Q9NZL9 | 37.551,60 | 100,00% | 5 | 6 | 8 | 0,16% | 23,70% | 334 |
|  | Pyruvate kinase PKM | P14618 | 57.937,50 | 100,00% | 4 | 4 | 4 | 0,08% | 8,66% | 531 |
|  | Aldo-keto reductase family 1 member B1 | P15121 | 35.853,90 | 100,00% | 5 | 5 | 7 | 0,14% | 23,40% | 316 |
|  | Pirin | O00625 | 32.113,70 | 100,00% | 3 | 3 | 3 | 0,06% | 11,00% | 290 |
|  | Malate dehydrogenase, mitochondrial | P40926 | 35.503,70 | 100,00% | 3 | 3 | 3 | 0,06% | 9,17% | 338 |
|  | Annexin A2 | P07355 | 38.606,10 | 100,00% | 20 | 21 | 22 | 0,45% | 51,30% | 339 |
|  | Poly(rC)-binding protein 1 | Q15365 | 37.498,20 | 100,00% | 1 | 1 | 4 | 0,08% | 12,60% | 356 |
|  | Aldo-keto reductase family 1 member A1 | P14550 | 36.573,60 | 100,00% | 8 | 8 | 12 | 0,24% | 31,10% | 325 |
|  | Glyceraldehyde-3-phosphate dehydrogenase | P04406 | 36.053,40 | 100,00% | 5 | 7 | 9 | 0,18% | 20,60% | 335 |
|  | LIM and SH3 domain protein 1 | Q14847 | 29.716,80 | 100,00% | 24 | 39 | 78 | 1,59% | 58,20% | 261 |
|  | Tropomyosin alpha-4 chain | P67936 | 28.522,40 | 99,00% | 0 | 0 | 9 | 0,18% | 29,40% | 248 |
|  | Sialic acid synthase | Q9NR45 | 40.307,90 | 100,00% | 4 | 4 | 5 | 0,10% | 16,40% | 359 |
|  | Annexin A1 | P04083 | 38.715,90 | 100,00% | 27 | 38 | 52 | 1,06% | 65,90% | 346 |
|  | OCIA domain-containing protein 1 | Q9NX40 | 27.626,80 | 100,00% | 3 | 4 | 6 | 0,12% | 9,80% | 245 |
|  | Isobutyryl-CoA dehydrogenase, mitochondrial | Q9UKU7 | 45.070,10 | 100,00% | 6 | 7 | 7 | 0,14% | 15,90% | 415 |
|  | Fructose-bisphosphate aldolase C | P09972 | 39.456,20 | 100,00% | 3 | 3 | 3 | 0,06% | 10,40% | 364 |
|  | Fructose-bisphosphate aldolase A | P04075 | 39.420,60 | 100,00% | 3 | 4 | 4 | 0,08% | 7,97% | 364 |
|  | Transaldolase | P37837 | 37.541,70 | 100,00% | 16 | 20 | 22 | 0,45% | 42,40% | 337 |
|  | Glyoxylate reductase/hydroxypyruvate reductase | Q9UBQ7 | 35.669,10 | 100,00% | 8 | 9 | 10 | 0,20% | 24,40% | 328 |
|  | 3-hydroxyisobutyryl-CoA hydrolase, mitochondrial | Q6NVY1 | 43.484,00 | 100,00% | 5 | 7 | 7 | 0,14% | 14,80% | 386 |
|  | Isoform 2 of Tropomyosin alpha-3 chain | P06753-2 | 29.033,30 | 100,00% | 8 | 9 | 14 | 0,29% | 49,20% | 248 |
|  | Poly(rC)-binding protein 2 | Q15366 | 38.580,90 | 99,90% | 1 | 1 | 4 | 0,08% | 12,30% | 365 |
|  | PDZ and LIM domain protein 4 | P50479 | 35.398,20 | 100,00% | 10 | 13 | 18 | 0,37% | 42,40% | 330 |
|  | Oxygen-dependent coproporphyrinogen-III oxidase, mitochondrial | P36551 | 50.152,00 | 100,00% | 9 | 11 | 11 | 0,22% | 24,90% | 454 |
|  | Endoplasmic reticulum chaperone BiP | P11021 | 72.334,70 | 100,00% | 3 | 3 | 3 | 0,06% | 7,03% | 654 |
|  | Serine/arginine-rich splicing factor 1 | Q07955 | 27.745,10 | 100,00% | 5 | 5 | 5 | 0,10% | 17,30% | 248 |
|  | Isoform 2 of Heterogeneous nuclear ribonucleoprotein D-like | O14979-2 | 33.589,60 | 100,00% | 18 | 28 | 39 | 0,79% | 45,50% | 301 |
| Spot 279 | Phosphomevalonate kinase | Q15126 | 21.995,00 | 100,00% | 4 | 4 | 4 | 0,13% | 18,80% | 192 |
|  | Intraflagellar transport protein 27 homolog | Q9BW83 | 20.480,50 | 100,00% | 3 | 3 | 5 | 0,16% | 17,70% | 186 |
|  | Isoform 2 of Core-binding factor subunit beta | Q13951-2 | 21.991,70 | 100,00% | 5 | 6 | 8 | 0,25% | 29,40% | 187 |
|  | Transgelin-2 | P37802 | 22.391,90 | 100,00% | 4 | 4 | 4 | 0,13% | 22,60% | 199 |
|  | Dynactin subunit 3 | O75935 | 21.119,80 | 100,00% | 3 | 3 | 4 | 0,13% | 13,40% | 186 |
|  | Adenine phosphoribosyltransferase | P07741 | 19.608,50 | 100,00% | 6 | 7 | 20 | 0,63% | 31,70% | 180 |
|  | Peroxiredoxin-2 | P32119 | 21.892,40 | 100,00% | 10 | 15 | 64 | 2,02% | 40,90% | 198 |
| Spot 283 | Glutathione S-transferase P1 | P09211 | 23.356,70 | 100,00% | 4 | 4 | 6 | 7,59% | 22,90% | 210 |
| Spot 299 | TAR DNA-binding protein 43 | A0A087WX29 | 44.739,70 | 100,00% | 4 | 5 | 5 | 0,12% | 10,60% | 414 |
|  | Serpin B5 | P36952 | 42.101,80 | 100,00% | 7 | 9 | 12 | 0,28% | 24,30% | 375 |
|  | 40S ribosomal protein SA | P08865 | 32.854,10 | 100,00% | 3 | 3 | 3 | 0,07% | 13,90% | 295 |
|  | Aspartate aminotransferase, mitochondrial | P00505 | 47.518,60 | 100,00% | 5 | 5 | 5 | 0,12% | 13,70% | 430 |
|  | Transcriptional activator protein Pur-alpha | Q00577 | 34.911,10 | 100,00% | 10 | 15 | 15 | 0,35% | 27,00% | 322 |
|  | [3-methyl-2-oxobutanoate dehydrogenase [lipoamide]] kinase, mitochondrial | O14874 | 46.362,40 | 100,00% | 3 | 3 | 3 | 0,07% | 8,98% | 412 |
|  | Mitotic checkpoint protein BUB3 | O43684 | 36.954,50 | 100,00% | 4 | 5 | 5 | 0,12% | 14,60% | 326 |
|  | Aminoacylase-1 | A0A1B0GU86 | 45.884,30 | 100,00% | 14 | 23 | 32 | 0,75% | 31,40% | 408 |
|  | Receptor tyrosine-protein kinase erbB-2 | P04626 | 137.910,50 | 100,00% | 3 | 4 | 6 | 0,14% | 3,90% | 1255 |
|  | Endophilin-B1 | Q9Y371 | 40.798,00 | 100,00% | 3 | 3 | 3 | 0,07% | 10,40% | 365 |
|  | Annexin A2 | P07355 | 38.606,10 | 100,00% | 8 | 8 | 8 | 0,19% | 30,40% | 339 |
|  | Transcription factor jun-B | P17275 | 35.879,80 | 100,00% | 6 | 6 | 6 | 0,14% | 29,10% | 347 |
|  | Actin, cytoplasmic 2 | P63261 | 41.793,90 | 100,00% | 1 | 1 | 49 | 1,15% | 54,40% | 375 |
|  | Poly(rC)-binding protein 1 | Q15365 | 37.498,20 | 100,00% | 4 | 4 | 9 | 0,21% | 30,10% | 356 |
|  | Stomatin-like protein 2, mitochondrial | Q9UJZ1 | 38.534,50 | 100,00% | 3 | 3 | 3 | 0,07% | 15,20% | 356 |
|  | Adenosylhomocysteinase | P23526 | 47.717,10 | 100,00% | 6 | 7 | 7 | 0,16% | 16,70% | 432 |
|  | Succinate--CoA ligase [ADP-forming] subunit beta, mitochondrial | Q9P2R7 | 50.318,10 | 100,00% | 3 | 3 | 3 | 0,07% | 6,26% | 463 |
|  | Isoform 2 of Heterogeneous nuclear ribonucleoprotein A/B | Q99729-2 | 30.303,00 | 100,00% | 9 | 13 | 15 | 0,35% | 22,90% | 280 |
|  | Glyceraldehyde-3-phosphate dehydrogenase | P04406 | 36.053,40 | 100,00% | 4 | 4 | 5 | 0,12% | 17,30% | 335 |
|  | Septin-2 | Q15019 | 41.488,20 | 100,00% | 8 | 10 | 15 | 0,35% | 31,00% | 361 |
|  | Leukocyte elastase inhibitor | P30740 | 42.743,80 | 100,00% | 24 | 34 | 42 | 0,98% | 50,70% | 379 |
|  | DnaJ homolog subfamily B member 11 | Q9UBS4 | 40.514,60 | 100,00% | 8 | 10 | 13 | 0,30% | 26,80% | 358 |
|  | Actin, aortic smooth muscle | P62736 | 42.020,10 | 100,00% | 2 | 2 | 34 | 0,80% | 33,20% | 377 |
|  | Fructose-bisphosphate aldolase C | P09972 | 39.456,20 | 100,00% | 10 | 11 | 11 | 0,26% | 23,40% | 364 |
|  | Heterogeneous nuclear ribonucleoprotein D0 | Q14103 | 38.434,50 | 100,00% | 2 | 2 | 3 | 0,07% | 7,32% | 355 |
|  | Fructose-bisphosphate aldolase A | P04075 | 39.420,60 | 100,00% | 16 | 21 | 28 | 0,66% | 48,40% | 364 |
|  | Transaldolase | P37837 | 37.541,70 | 100,00% | 3 | 3 | 3 | 0,07% | 11,60% | 337 |
|  | 26S proteasome regulatory subunit 10B | A0A087X2I1 | 44.174,90 | 100,00% | 4 | 4 | 4 | 0,09% | 13,10% | 389 |
|  | GDP-D-glucose phosphorylase 1 | Q6ZNW5 | 42.363,00 | 100,00% | 3 | 3 | 3 | 0,07% | 10,60% | 385 |
|  | Macrophage-capping protein | P40121 | 38.498,90 | 100,00% | 20 | 32 | 51 | 1,19% | 45,40% | 348 |
|  | Poly(rC)-binding protein 2 | F8VZX2 | 38.580,90 | 100,00% | 2 | 2 | 7 | 0,16% | 18,10% | 365 |
|  | Actin-like protein 8 | Q9H568 | 41.360,30 | 100,00% | 10 | 15 | 16 | 0,37% | 32,20% | 366 |
|  | MYG1 exonuclease | Q9HB07 | 42.477,90 | 100,00% | 2 | 2 | 9 | 0,21% | 28,70% | 376 |
|  | Phosphoglycerate kinase 1 | P00558 | 44.615,30 | 100,00% | 15 | 23 | 29 | 0,68% | 42,20% | 417 |
|  | Proteasomal ATPase-associated factor 1 | Q9BRP4 | 42.190,40 | 100,00% | 3 | 3 | 3 | 0,07% | 7,65% | 392 |
|  | Heterogeneous nuclear ribonucleoproteins C1/C2 | P07910 | 33.670,50 | 100,00% | 4 | 4 | 4 | 0,09% | 15,70% | 306 |
|  | RNA 3'-terminal phosphate cyclase | O00442 | 39.337,50 | 100,00% | 3 | 3 | 3 | 0,07% | 10,70% | 366 |
|  | PDZ domain-containing protein GIPC1 | O14908 | 36.050,60 | 100,00% | 3 | 3 | 3 | 0,07% | 12,90% | 333 |
|  | Actin, cytoplasmic 1 | P60709 | 41.737,80 | 100,00% | 1 | 1 | 50 | 1,17% | 54,40% | 375 |
|  | MYG1 exonuclease | F8VQQ3 | 33.820,00 | 100,00% | 1 | 1 | 8 | 0,19% | 30,60% | 301 |
|  | 3'(2'),5'-bisphosphate nucleotidase 1 | O95861 | 33.392,70 | 100,00% | 6 | 7 | 7 | 0,16% | 21,40% | 308 |
| Spot 303 | 14-3-3 protein sigma | P31947 | 27.774,80 | 100,00% | 7 | 11 | 17 | 0,40% | 35,50% | 248 |
|  | Prohibitin | P35232 | 29.804,60 | 100,00% | 3 | 3 | 3 | 0,07% | 11,80% | 272 |
|  | Tropomyosin alpha-4 chain | A0A2R8Y5V9 | 28.609,80 | 100,00% | 2 | 2 | 86 | 2,00% | 85,50% | 248 |
|  | Delta(3,5)-Delta(2,4)-dienoyl-CoA isomerase, mitochondrial | Q13011 | 35.816,20 | 100,00% | 3 | 3 | 3 | 0,07% | 12,20% | 328 |
|  | Cathepsin B | P07858 | 37.821,20 | 100,00% | 3 | 3 | 3 | 0,07% | 6,49% | 339 |
|  | 14-3-3 protein zeta/delta | P63104 | 27.745,90 | 100,00% | 4 | 5 | 8 | 0,19% | 38,40% | 245 |
|  | Heme-binding protein 2 | Q9Y5Z4 | 22.875,70 | 100,00% | 5 | 5 | 7 | 0,16% | 25,40% | 205 |
|  | Elongation factor 1-beta | P24534 | 24.764,20 | 100,00% | 7 | 9 | 23 | 0,54% | 31,10% | 225 |
|  | Serine/arginine-rich splicing factor 2 | Q01130 | 25.477,10 | 100,00% | 3 | 3 | 3 | 0,07% | 18,60% | 221 |
|  | 14-3-3 protein gamma | P61981 | 28.303,10 | 100,00% | 3 | 3 | 7 | 0,16% | 27,50% | 247 |
|  | Tumor protein D54 | O43399 | 22.237,90 | 100,00% | 1 | 1 | 7 | 0,16% | 42,20% | 206 |
|  | Tropomyosin 1 (Alpha), isoform CRA_b | H0YKJ4 | 28.598,70 | 100,00% | 2 | 2 | 47 | 1,09% | 67,70% | 248 |
|  | ADP-sugar pyrophosphatase | Q9UKK9 | 24.327,80 | 100,00% | 4 | 4 | 5 | 0,12% | 17,40% | 219 |
|  | 14-3-3 protein beta/alpha | P31946 | 28.083,10 | 100,00% | 2 | 2 | 6 | 0,14% | 22,00% | 246 |
|  | Chloride intracellular channel protein 1 | O00299 | 26.923,30 | 100,00% | 7 | 8 | 10 | 0,23% | 29,00% | 241 |
|  | 14-3-3 protein epsilon | P62258 | 29.175,00 | 100,00% | 12 | 16 | 23 | 0,54% | 56,10% | 255 |
|  | Tropomyosin alpha-1 chain | B7Z596 | 31.754,00 | 100,00% | 0 | 0 | 45 | 1,05% | 50,50% | 275 |
|  | Proteasome subunit alpha type-1 | P25786 | 29.556,00 | 100,00% | 7 | 8 | 10 | 0,23% | 27,00% | 263 |
|  | Tropomyosin alpha-4 chain | P67936 | 28.522,40 | 100,00% | 3 | 4 | 88 | 2,05% | 80,20% | 248 |
|  | Actin, aortic smooth muscle | P62736 | 42.020,10 | 100,00% | 2 | 2 | 7 | 0,16% | 22,50% | 377 |
|  | Microtubule-associated protein 1B | P46821 | 270.634,40 | 100,00% | 3 | 3 | 3 | 0,07% | 1,70% | 2468 |
|  | Annexin A4 | P09525 | 35.884,30 | 100,00% | 5 | 5 | 5 | 0,12% | 14,40% | 319 |
|  | Acidic leucine-rich nuclear phosphoprotein 32 family member A | P39687 | 28.586,10 | 100,00% | 7 | 8 | 9 | 0,21% | 20,10% | 249 |
|  | THO complex subunit 4 | Q86V81 | 26.888,70 | 100,00% | 8 | 11 | 20 | 0,47% | 20,60% | 257 |
|  | Elongation factor 1-delta | P29692 | 31.121,90 | 99,90% | 1 | 1 | 3 | 0,07% | 7,47% | 281 |
|  | Isoform 2 of Tropomyosin alpha-3 chain | P06753-2 | 29.033,30 | 100,00% | 27 | 42 | 112 | 2,60% | 91,10% | 248 |
|  | Cathepsin D | P07339 | 44.553,00 | 100,00% | 6 | 6 | 9 | 0,21% | 15,80% | 412 |
|  | Electron transfer flavoprotein subunit alpha, mitochondrial | P13804 | 35.080,10 | 100,00% | 3 | 3 | 3 | 0,07% | 12,30% | 333 |
|  | Isoform 2 of Tumor protein D54 | O43399-2 | 19.901,30 | 99,70% | 0 | 0 | 6 | 0,14% | 39,80% | 186 |
|  | Endoplasmic reticulum chaperone BiP | P11021 | 72.334,70 | 100,00% | 3 | 3 | 3 | 0,07% | 5,81% | 654 |
|  | Tubulin-folding cofactor B | A0A494C0X0 | 27.325,60 | 100,00% | 11 | 16 | 18 | 0,42% | 40,60% | 244 |
|  | Microtubule-associated protein RP/EB family member 1 | Q15691 | 29.999,50 | 100,00% | 5 | 7 | 7 | 0,16% | 25,00% | 268 |
|  | Ran-specific GTPase-activating protein | P43487 | 23.310,80 | 100,00% | 3 | 3 | 3 | 0,07% | 11,90% | 201 |
|  | Purine nucleoside phosphorylase | P00491 | 32.118,00 | 100,00% | 3 | 3 | 4 | 0,09% | 14,50% | 289 |
|  | Serine/arginine-rich splicing factor 1 | J3KTL2 | 27.745,10 | 100,00% | 4 | 4 | 4 | 0,09% | 17,30% | 248 |
|  | 14-3-3 protein theta | P27348 | 27.765,40 | 100,00% | 3 | 3 | 6 | 0,14% | 24,50% | 245 |
|  | Rho GDP-dissociation inhibitor 1 | P52565 | 23.207,50 | 100,00% | 3 | 5 | 6 | 0,14% | 22,10% | 204 |
|  | Histone H1.10 | Q92522 | 22.487,90 | 100,00% | 4 | 4 | 5 | 0,12% | 26,30% | 213 |
|  | Carbonyl reductase [NADPH] 1 | P16152 | 30.374,80 | 100,00% | 4 | 4 | 4 | 0,09% | 17,70% | 277 |
