# Supplementary material for: Evaluation of the gastrotolerability of ketoprofen, lysine, and gabapentin co-crystal administration in an in vitro model of gastric epithelium: a proteomic update
Source: PLoS One. 2025 Jul 29;20(7):e0328496. doi: 10.1371/journal.pone.0328496 (PMC12306739; doi:10.1371/journal.pone.0328496)

**S6 Fig.** **Gene Ontology (GO) enriched analysis on the identified proteins for Pathway terms.** GO/Pathway terms specific for representative genes. The bars represent the number of genes associated with the terms. The percentage of genes per term is shown as bar label (*p ≤ 0.05; **p < 0.001).


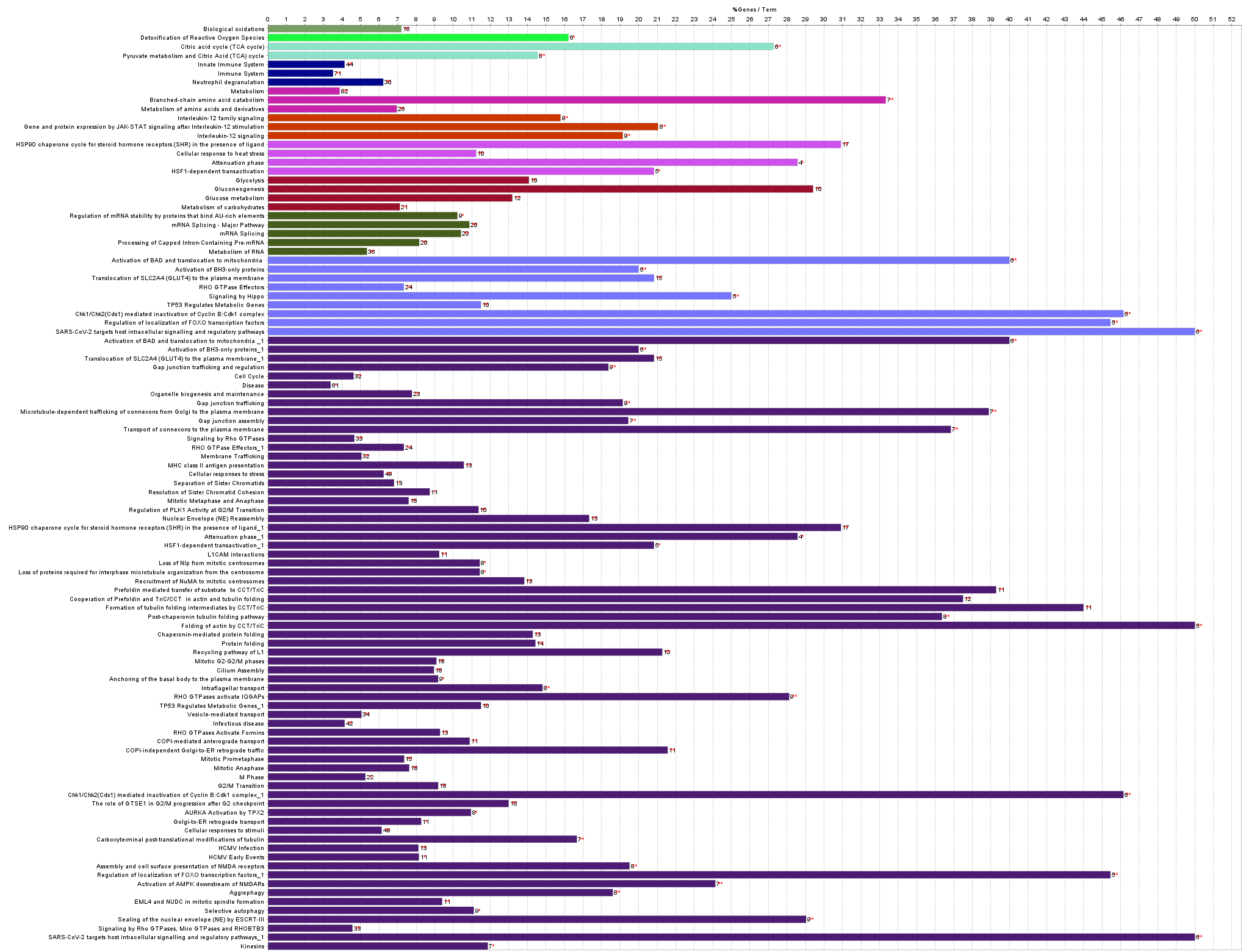

Supplement: S3 Fig — GO/Pathway terms specific for representative genes. The bars represent the number of genes associated with the terms. The percentage of genes per term is shown as bar label (*p ≤ 0.05; **p < 0.001). (DOCX) [file pone.0328496.s006.docx]
