# Supplementary material for: Evaluation of the gastrotolerability of ketoprofen, lysine, and gabapentin co-crystal administration in an in vitro model of gastric epithelium: a proteomic update
Source: PLoS One. 2025 Jul 29;20(7):e0328496. doi: 10.1371/journal.pone.0328496 (PMC12306739; doi:10.1371/journal.pone.0328496)

**S7 Fig.** **Effects of drug treatments in the leaky gut in vitro model.** Representative bright field pictures of untreated control N87 and ethanol-injured N87 treated 72 hours with different treatments. Bar= 400 μm.


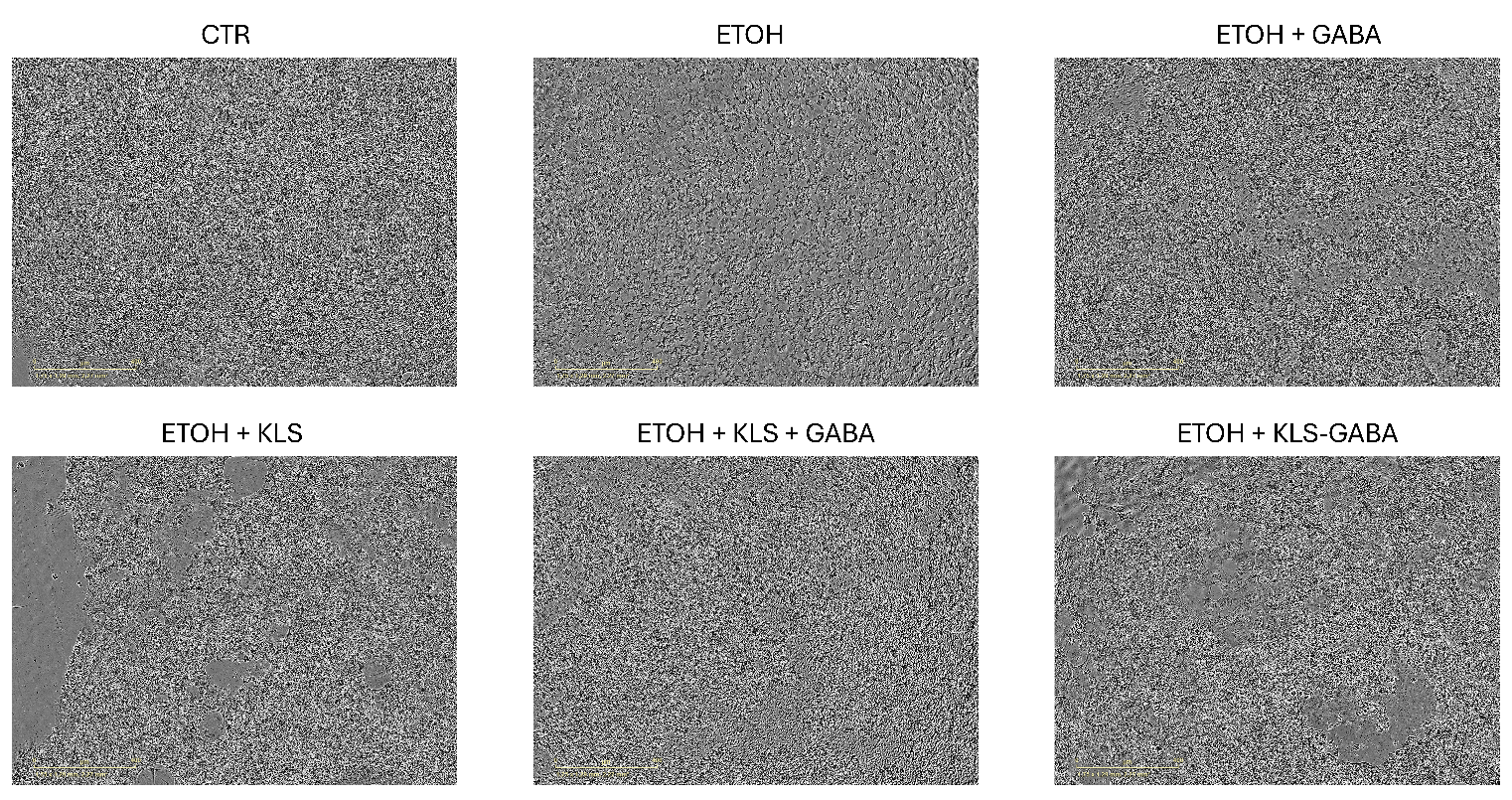

Supplement: S4 Fig — Representative bright field pictures of untreated control N87 and ethanol-injured N87 treated 72 hours with different treatments. Bar = 400 μm. (DOCX) [file pone.0328496.s007.docx]
