# Supplementary material for: Evaluation of the gastrotolerability of ketoprofen, lysine, and gabapentin co-crystal administration in an in vitro model of gastric epithelium: a proteomic update
Source: PLoS One. 2025 Jul 29;20(7):e0328496. doi: 10.1371/journal.pone.0328496 (PMC12306739; doi:10.1371/journal.pone.0328496)
Supplement: S5 Fig — Raw images of 2DE gels divided by replicates. For Figure 1 panel the first replicate was used. (PDF) [file pone.0328496.s008.pdf]

S8_raw_images. Figure 1. Raw images of 2DE gels divided by replicates. For Figure 1 panel the first replicate was used.

**FIRST REPLICATE - 2DE**			
**CTR**	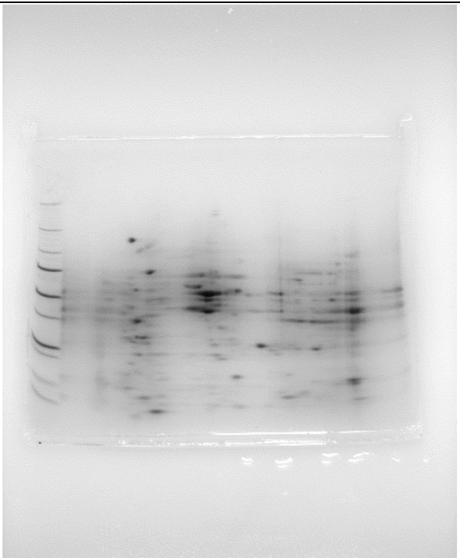	**ETOH**	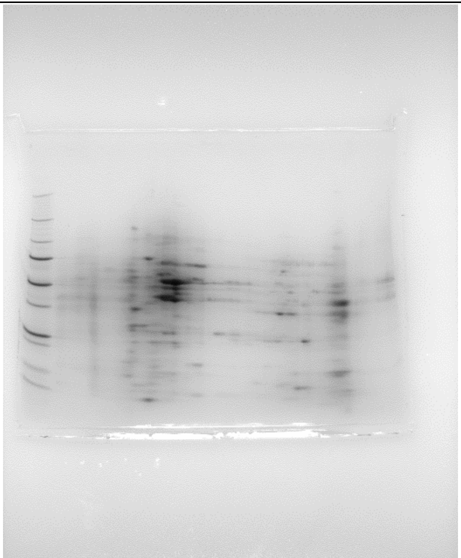
**ETOH+GABA**	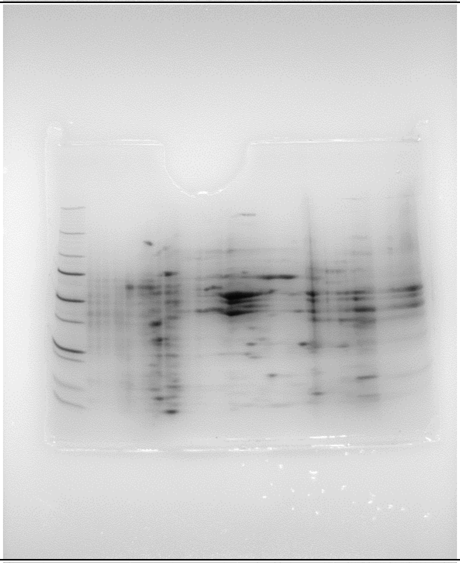	**ETOH+KLS**	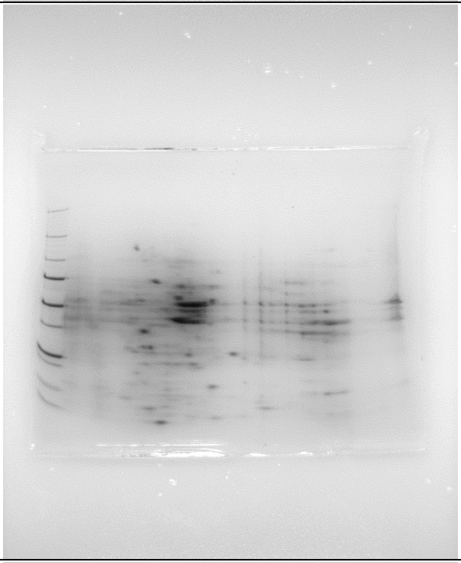
**ETOH+KLS+GABA**	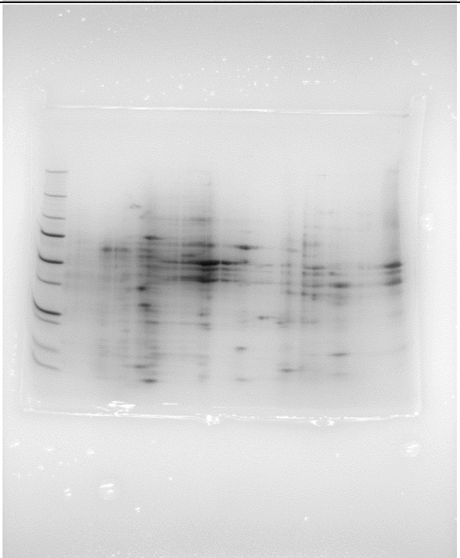	**ETOH+KLS-GABA**	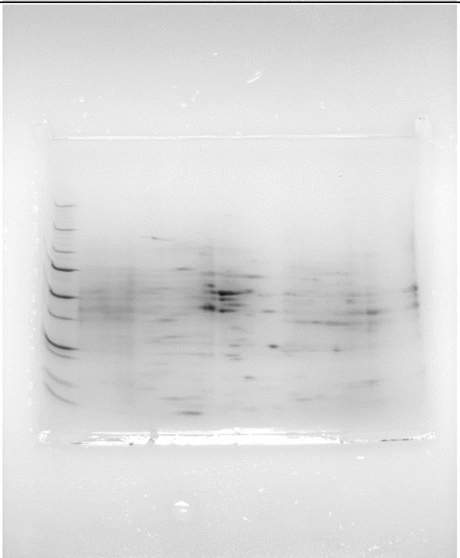

SECOND REPLICATE - 2DE			
CTR	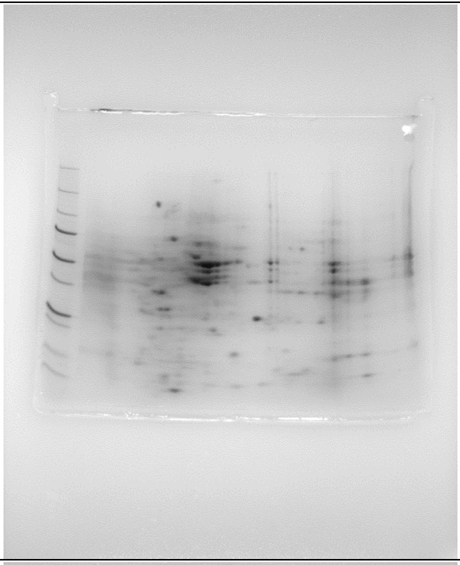	ETOH	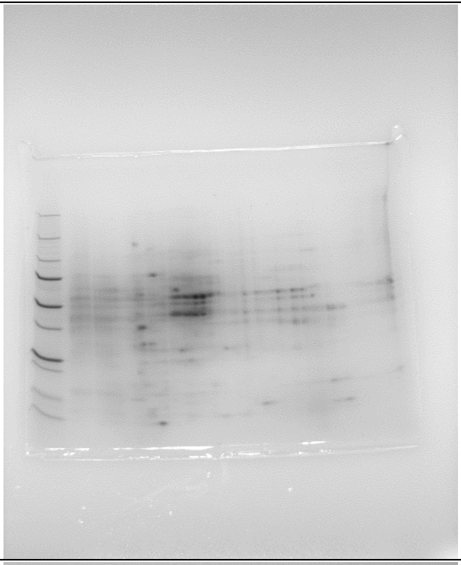
ETOH+GABA	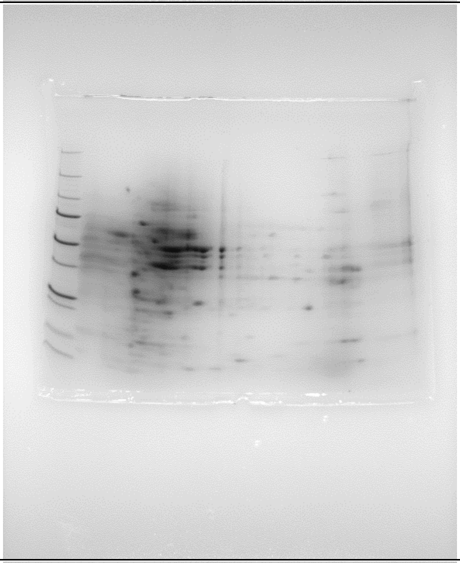	ETOH+KLS	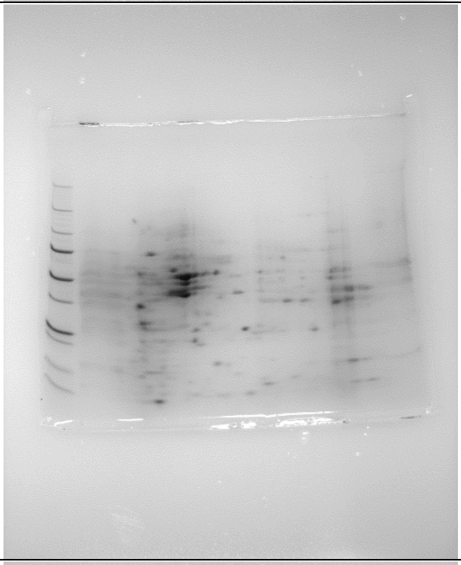
ETOH+KLS+GABA	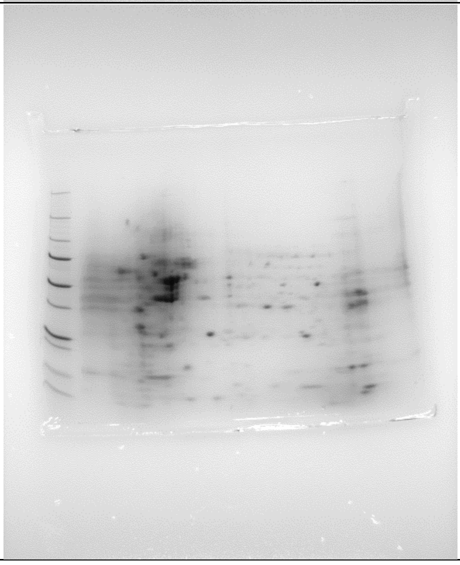	ETOH+KLS-GABA	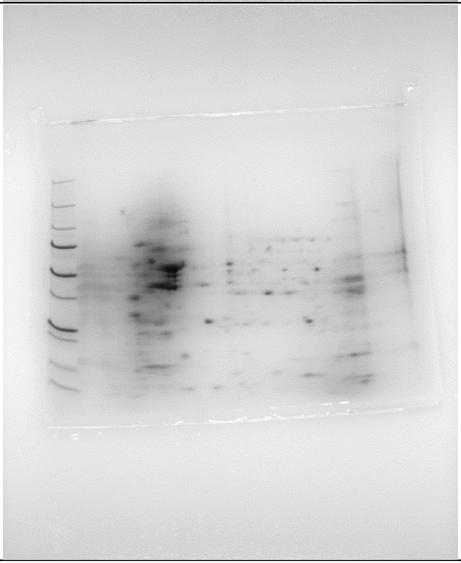

THIRD REPLICATE - 2DE			
**CTR**	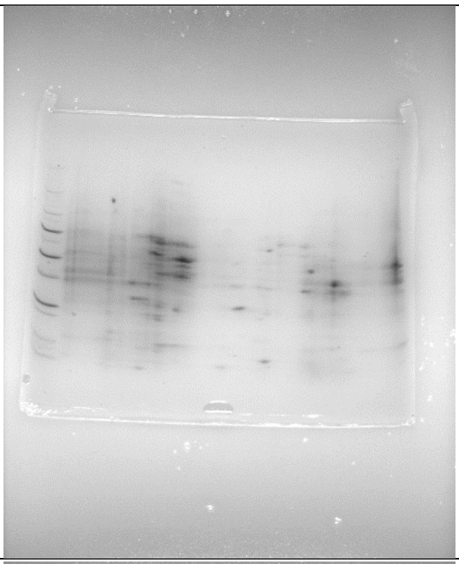	**ETOH**	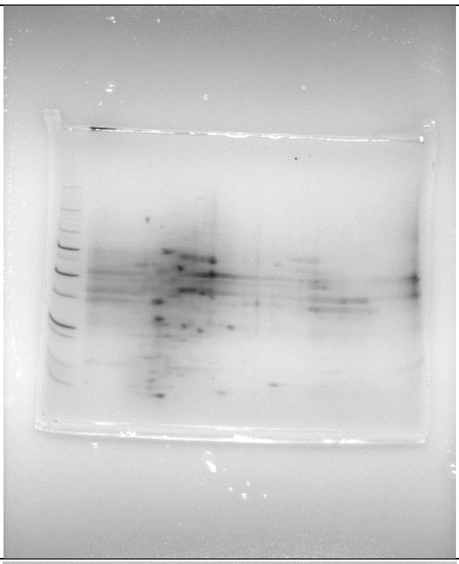
**ETOH+GABA**	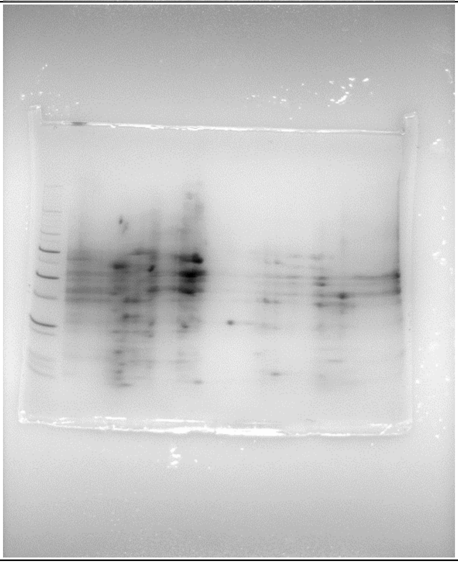	**ETOH+KLS**	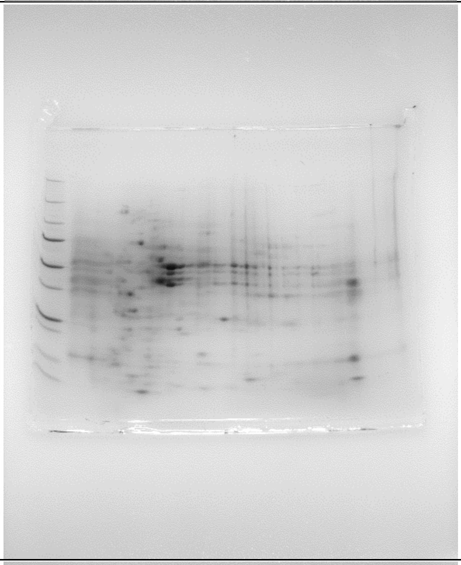
**ETOH+KLS+GABA**	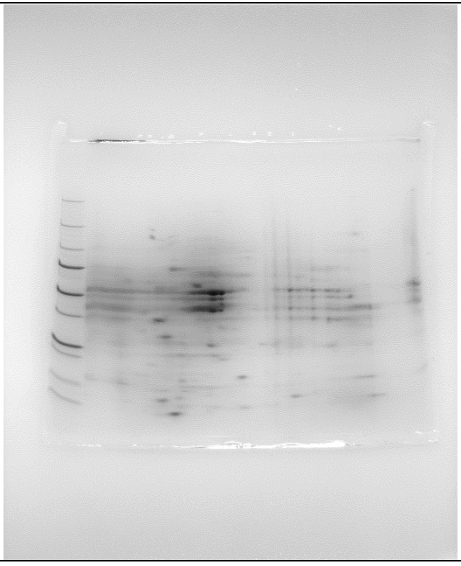	**ETOH+KLS-GABA**	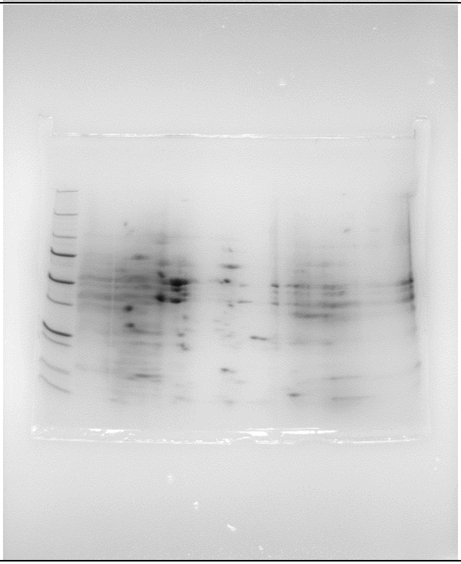
