# Supplementary material for: Evaluation of the gastrotolerability of ketoprofen, lysine, and gabapentin co-crystal administration in an in vitro model of gastric epithelium: a proteomic update
Source: PLoS One. 2025 Jul 29;20(7):e0328496. doi: 10.1371/journal.pone.0328496 (PMC12306739; doi:10.1371/journal.pone.0328496)

S9_raw_images. Figure 3.2. Raw images of Western Blotting analysis for GSTP1. On the left images, the highlighted sections show bands for GSTP1. On the right, the highlighted sections show the reference on which GSTP1 data were normalized. The panel in figure 3.2 in the manuscript shows sections of the figure here highlighted in a green square.

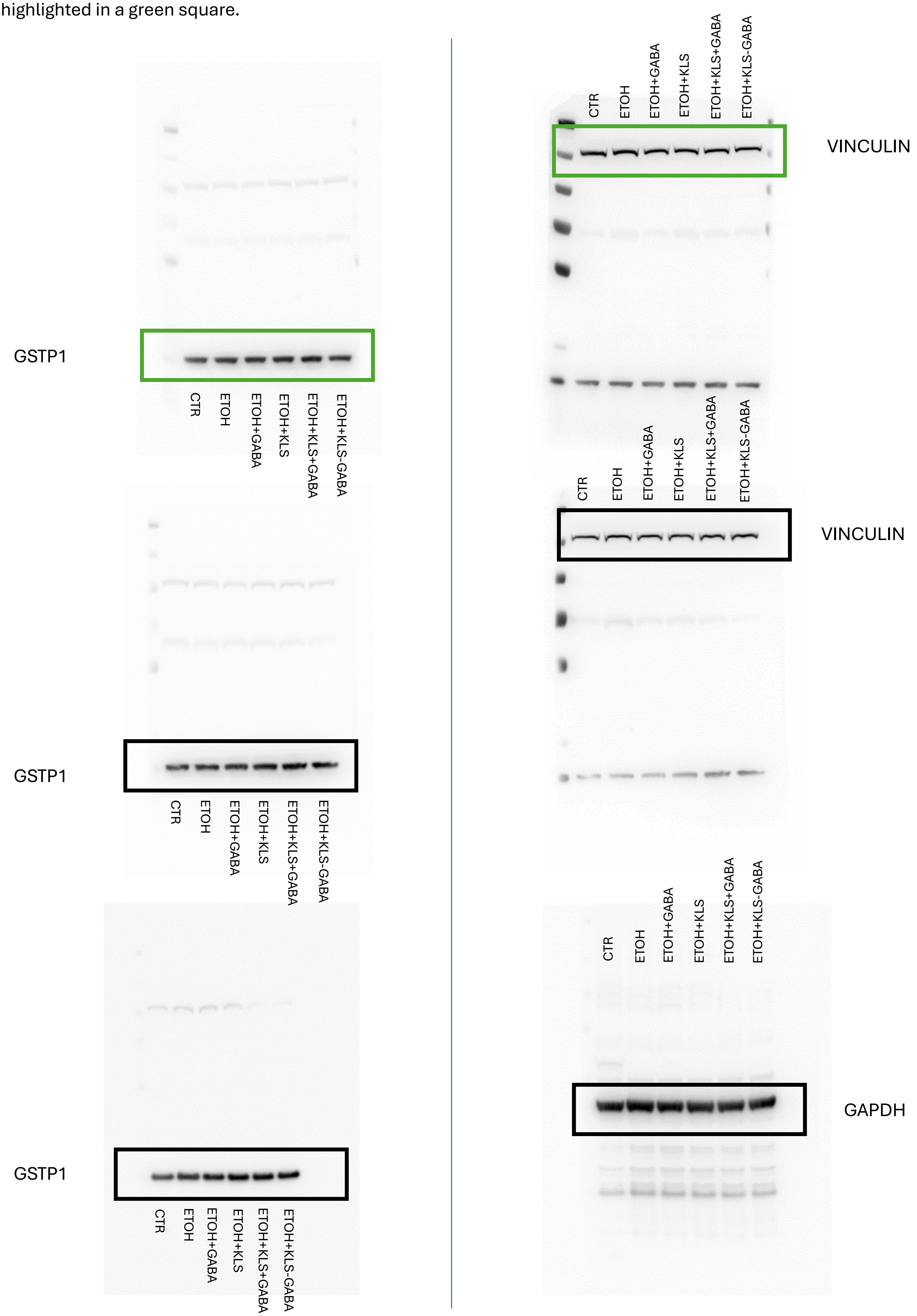

Supplement: S6 Fig — On the left images, the highlighted sections show bands for GSTP1. On the right, the highlighted sections show the reference on which GSTP1 data were normalized. The panel in figure 3.2 in the manuscript shows sections of the figure here highlighted in a green square. (PDF) [file pone.0328496.s009.pdf]
