# Supplementary material for: Evaluation of the gastrotolerability of ketoprofen, lysine, and gabapentin co-crystal administration in an in vitro model of gastric epithelium: a proteomic update
Source: PLoS One. 2025 Jul 29;20(7):e0328496. doi: 10.1371/journal.pone.0328496 (PMC12306739; doi:10.1371/journal.pone.0328496)
Supplement: S7 Fig — Representative bright field pictures of untreated control N87 and ethanol-injured N87 treated 72 hours with different treatments. Bar = 400 μm. (PDF) [file pone.0328496.s010.pdf]

S10_raw_images. Figure S7. Effects of drug treatments in the leaky gut in vitro model. Representative bright field pictures of untreated control N87 and ethanol-injured N87 treated 72 hours with different treatments. Bar= 400 μm.

***IN VITRO* MODEL BRIGHT FIELD PICTURES**			
**CTR**	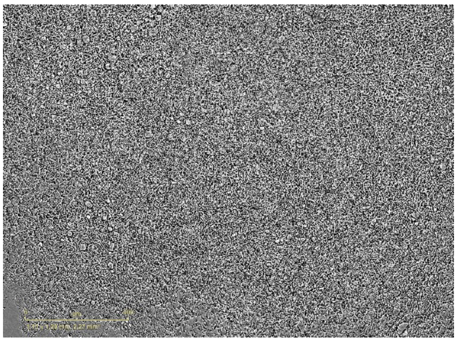	**ETOH**	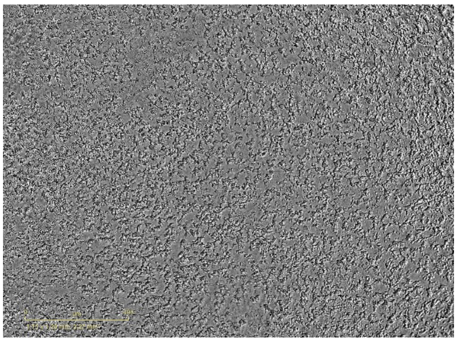
**ETOH+GABA**	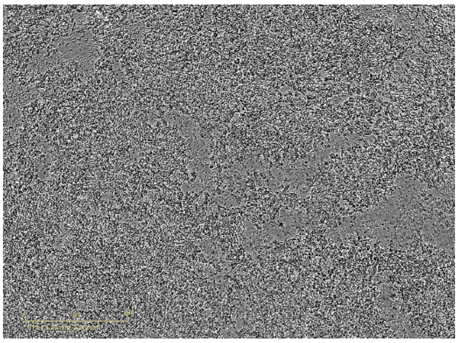	**ETOH+KLS**	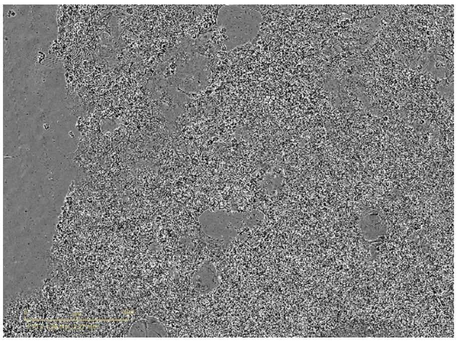
**ETOH+KLS+GABA**	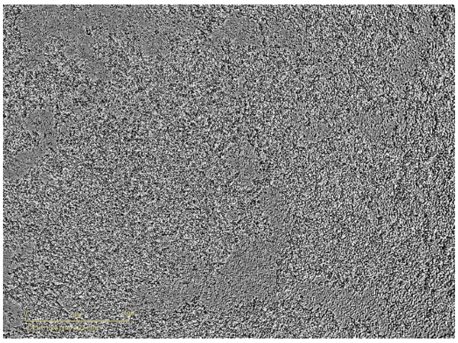	**ETOH+KLS-GABA**	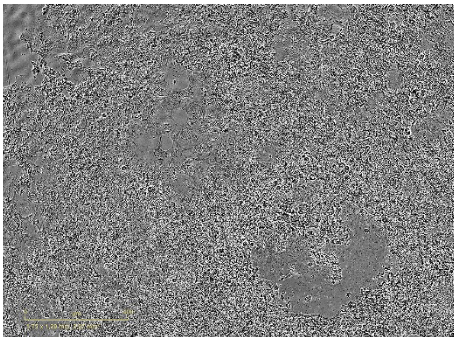
