# Supplementary material for: Evaluation of the gastrotolerability of ketoprofen, lysine, and gabapentin co-crystal administration in an in vitro model of gastric epithelium: a proteomic update
Source: PLoS One. 2025 Jul 29;20(7):e0328496. doi: 10.1371/journal.pone.0328496 (PMC12306739; doi:10.1371/journal.pone.0328496)
Supplement: Peer review history

# Peer review: Decision Letter 0

**DOI:** 10.1371/journal.pone.0328496.r001

**Authors:** Anisha DSouza

Dear Dr. Cimini,

Thank you for submitting your manuscript to PLOS ONE. After careful consideration, we feel that it has merit but does not fully meet PLOS ONE’s publication criteria as it currently stands. Therefore, we invite you to submit a revised version of the manuscript that addresses the points raised during the review process.

Please submit your revised manuscript by Jun 22 2025 11:59PM. If you will need more time than this to complete your revisions, please reply to this message or contact the journal office at plosone@plos.org . When you're ready to submit your revision, log on to https://www.editorialmanager.com/pone/ and select the 'Submissions Needing Revision' folder to locate your manuscript file.

- A rebuttal letter that responds to each point raised by the academic editor and reviewer(s). You should upload this letter as a separate file labeled 'Response to Reviewers'.
- A marked-up copy of your manuscript that highlights changes made to the original version. You should upload this as a separate file labeled 'Revised Manuscript with Track Changes'.
- An unmarked version of your revised paper without tracked changes. You should upload this as a separate file labeled 'Manuscript'.

If you would like to make changes to your financial disclosure, please include your updated statement in your cover letter. Guidelines for resubmitting your figure files are available below the reviewer comments at the end of this letter.

If applicable, we recommend that you deposit your laboratory protocols in protocols.io to enhance the reproducibility of your results. Protocols.io assigns your protocol its own identifier (DOI) so that it can be cited independently in the future. For instructions see: https://journals.plos.org/plosone/s/submission-guidelines#loc-laboratory-protocols . Additionally, PLOS ONE offers an option for publishing peer-reviewed Lab Protocol articles, which describe protocols hosted on protocols.io. Read more information on sharing protocols at https://plos.org/protocols?utm_medium=editorial-email&utm_source=authorletters&utm_campaign=protocols .

We look forward to receiving your revised manuscript.

Kind regards,

Anisha DSouza, Ph.D (Tech)

Academic Editor

PLOS ONE

Journal Requirements:

When submitting your revision, we need you to address these additional requirements.

1. Please ensure that your manuscript meets PLOS ONE's style requirements, including those for file naming. The PLOS ONE style templates can be found at

https://journals.plos.org/plosone/s/file?id=wjVg/PLOSOne_formatting_sample_main_body.pdf and

https://journals.plos.org/plosone/s/file?id=ba62/PLOSOne_formatting_sample_title_authors_affiliations.pdf

2. If any supporting files for review show as item type ‘other’ please change to item type ‘supporting info’ as the reviewer does not have access to these ’other’ files

3. Thank you for stating the following in the Competing Interests section: [Laura Brandolini, Andrea Aramini and Marcello Allegretti are employees of Dompe pharmaceutical Spa]. 

We note that you received funding from a commercial source: [Dompe pharmaceutical Spa]

Please provide an amended Competing Interests Statement that explicitly states this commercial funder, along with any other relevant declarations relating to employment, consultancy, patents, products in development, marketed products, etc.

Within this Competing Interests Statement, please confirm that this does not alter your adherence to all PLOS ONE policies on sharing data and materials by including the following statement: ""This does not alter our adherence to PLOS ONE policies on sharing data and materials.” (as detailed online in our guide for authors http://journals.plos.org/plosone/s/competing-interests).  If there are restrictions on sharing of data and/or materials, please state these. Please note that we cannot proceed with consideration of your article until this information has been declared.

Please include your amended Competing Interests Statement within your cover letter. We will change the online submission form on your behalf.

4. When completing the data availability statement of the submission form, you indicated that you will make your data available on acceptance. We strongly recommend all authors decide on a data sharing plan before acceptance, as the process can be lengthy and hold up publication timelines. Please note that, though access restrictions are acceptable now, your entire data will need to be made freely accessible if your manuscript is accepted for publication. This policy applies to all data except where public deposition would breach compliance with the protocol approved by your research ethics board. If you are unable to adhere to our open data policy, please kindly revise your statement to explain your reasoning and we will seek the editor's input on an exemption. Please be assured that, once you have provided your new statement, the assessment of your exemption will not hold up the peer review process.

5. PLOS requires an ORCID iD for the corresponding author in Editorial Manager on papers submitted after December 6th, 2016. Please ensure that you have an ORCID iD and that it is validated in Editorial Manager. To do this, go to ‘Update my Information’ (in the upper left-hand corner of the main menu), and click on the Fetch/Validate link next to the ORCID field. This will take you to the ORCID site and allow you to create a new iD or authenticate a pre-existing iD in Editorial Manager.

6. PLOS ONE now requires that authors provide the original uncropped and unadjusted images underlying all blot or gel results reported in a submission’s figures or Supporting Information files. This policy and the journal’s other requirements for blot/gel reporting and figure preparation are described in detail at https://journals.plos.org/plosone/s/figures#loc-blot-and-gel-reporting-requirements and https://journals.plos.org/plosone/s/figures#loc-preparing-figures-from-image-files. When you submit your revised manuscript, please ensure that your figures adhere fully to these guidelines and provide the original underlying images for all blot or gel data reported in your submission. See the following link for instructions on providing the original image data: https://journals.plos.org/plosone/s/figures#loc-original-images-for-blots-and-gels.  

In your cover letter, please note whether your blot/gel image data are in Supporting Information or posted at a public data repository, provide the repository URL if relevant, and provide specific details as to which raw blot/gel images, if any, are not available. Email us at plosone@plos.org if you have any questions.

Additional Editor Comments:

We have received the reviewer's comments now. Please revise it accordingly. Thanks!

[Note: HTML markup is below. Please do not edit.]

Reviewers' comments:

Reviewer's Responses to Questions

**Comments to the Author**

1. Is the manuscript technically sound, and do the data support the conclusions?

Reviewer #1: Yes

Reviewer #2: Yes

**********

2. Has the statistical analysis been performed appropriately and rigorously?

Reviewer #1: Yes

Reviewer #2: Yes

**********

3. Have the authors made all data underlying the findings in their manuscript fully available?

The PLOS Data policy

Reviewer #1: Yes

Reviewer #2: Yes

**********

4. Is the manuscript presented in an intelligible fashion and written in standard English?

Reviewer #1: Yes

Reviewer #2: Yes

**********

Reviewer #1: The authors should explain the following points and consider the following revisions.

1) Why do the authors believe that the pharmaceutical properties of KLS+GABA are different than KLS-GABA when both are dissolved and in solution during cellular experiments. The cocrystal form of KLS-GABA is irrelevant if its dissolved in solution, presumably in solution you would expect KLS-GABA and KLS+GABA to give similar outcomes. Can the authors further explain this ?

2) Can the authors explain what they mean by “factors and mechanisms involved in cocrystal effects” – once the crystal is dissolved and the drugs are in solution, it should be no different than KLS+GABA.

3) The authors should clearly highlight the drawbacks of 2DE like limited detection of low-abundance or hydrophobic proteins, and difficulty in resolving post-translational modifications, possibly leading to incomplete proteome characterization.

4) Can the authors highlight potential next steps to confirm the role of oxidative stress pathways ? ROS measurements ? Antioxidant Enzyme Activity ?

5) The Ethanol-Induced Injury Model has many limitations they do not fully replicate human gastric ulceration complexity, which includes factors like H. pylori infection and chronic NSAID use. Can the authors replicate this in-vitro and study its effects through a proteomic analysis.

Reviewer #2: Reviewer comments:

1. Figure 1- Please include molecular weight for marker in the figure.

2. Figure 3.2- Please include molecular weight for marker in the figure.

3. In depth in vitro studies will be necessary to establish the model and to check its effect. Can the authors include figures of cell line model in supplement figure to show the effect of co-crystal

**********

what does this mean? ). If published, this will include your full peer review and any attached files.

If you choose “no”, your identity will remain anonymous but your review may still be made public.

**Do you want your identity to be public for this peer review?** For information about this choice, including consent withdrawal, please see our Privacy Policy

Reviewer #1: No

Reviewer #2: No

**********

[NOTE: If reviewer comments were submitted as an attachment file, they will be attached to this email and accessible via the submission site. Please log into your account, locate the manuscript record, and check for the action link "View Attachments". If this link does not appear, there are no attachment files.]

While revising your submission, please upload your figure files to the Preflight Analysis and Conversion Engine (PACE) digital diagnostic tool, https://pacev2.apexcovantage.com/ . PACE helps ensure that figures meet PLOS requirements. To use PACE, you must first register as a user. Registration is free. Then, login and navigate to the UPLOAD tab, where you will find detailed instructions on how to use the tool. If you encounter any issues or have any questions when using PACE, please email PLOS at figures@plos.org . Please note that Supporting Information files do not need this step.

# Author comment: Author response to Decision Letter 1

**DOI:** 10.1371/journal.pone.0328496.r002

20 Jun 2025

June 16th, 2025

To: Editorial office

Dear Editors,

We are glad to receive your editorial decision that our manuscript

PONE-D-25-12165 has been provisionally accepted for publication pending satisfactory completion of some concerns. Please find below my point-by-point responses to the comments, which have been reflected in the revised manuscript (track changes):

Reviewer 1

The authors should explain the following points and consider the following revisions.

Response: We would like to thank Reviewer 1 for the comments provided that helped to improve our article. We tried to address all the points raised.

Comments

1. Why do the authors believe that the pharmaceutical properties of KLS+GABA are different than KLS-GABA when both are dissolved and in solution during cellular experiments. The cocrystal form of KLS-GABA is irrelevant if its dissolved in solution, presumably in solution you would expect KLS-GABA and KLS+GABA to give similar outcomes. Can the authors further explain this ?

Response: We appreciate the reviewer’s insightful comment and the opportunity to clarify this point. While we fully agree that, at equilibrium, both the physical mixture (KLS + GABA) and the KLS-GABA cocrystal ultimately yield the same molecular species in solution, a growing body of literature supports the view that solid-state properties can influence early pharmacological responses even under in vitro conditions. This is particularly relevant in cellular systems where exposure dynamics—not only final concentrations—play a key role in modulating biological effects.

In our specific model of epithelial barrier dysfunction (leaky gut), transient solubility behavior, supersaturation effects, and local microenvironmental conditions at the apical surface are crucial for determining cellular uptake, barrier modulation, and inflammatory signaling. These early-phase physicochemical events are not fully captured by assuming a uniform solution state.

As demonstrated in the study by Aramini et al. (https://doi.org/10.1016/j.biopha.2023.114845), the KLS-GABA cocrystal exhibited improved gastric solubility and a distinct dissolution profile compared to the individual components. The authors observed supersaturation phenomena, which resulted in a more rapid and sustained release of the active agents, contributing to enhanced pharmacological efficacy and tolerability. The described phenomena are functionally and mechanistically linked to solute–solvent interactions and coordinated molecular release, both of which are integral to the concept of solvation kinetics.

These findings are further supported by other reports showing that co-crystals can maintain transient non-equilibrium conditions in the immediate microenvironment—even in buffered, well-mixed systems—potentially affecting how drug molecules interact with cell membranes, transporters, and receptors. This is particularly important in monolayer-based in vitro models, where cellular responses are sensitive to changes in apical concentration, polarity, and permeability gradients.

Considering this, we argue that the pharmaceutical behavior of the cocrystal cannot be considered irrelevant, even in in vitro settings. The kinetic and spatial aspects of release from the cocrystal matrix can result in differential modulation of epithelial responses, as reflected in our proteomic and functional data. We now added this information in the manuscript.

2. Can the authors explain what they mean by “factors and mechanisms involved in cocrystal effects” – once the crystal is dissolved and the drugs are in solution, it should be no different than KLS+GABA.

Response: We thank the reviewer for the opportunity to clarify this. By “factors and mechanisms involved in cocrystal effects,” we refer to the influence of solid-state properties on dissolution kinetics, supersaturation behavior, and local solvation dynamics, particularly during the initial phase of drug exposure.

As discussed in Comment 1, and supported by Aramini et al. (https://doi.org/10.1016/j.biopha.2023.114845), the KLS-GABA cocrystal exhibited enhanced solubility and distinct supersaturation profiles compared to the physical mixture. These early, non-equilibrium phenomena can create localized concentration gradients at the apical surface of epithelial monolayers, affecting membrane interaction, transporter engagement, and barrier response.

To further investigate the significance of the supersaturation phenomenon, we evaluated the 3D aggregation state of KLS, KLS-GABA and KLS+GABA solutions and compared both the number and shape using an Ipac 2 image analyzer (Occhio s.a.). With KLS and KLS+GABA solutions, we observed the presence of spherical aggregates with an average inner diameter ranging from 17.56 to 86.12 μm and from 21.40 up to 54.75 μm, respectively, and an average concentration of particles/ μL of 363 and 17, respectively (Fig. 1B). In contrast, analysis of KLS-GABA co-crystal showed a background that was similar to that of milliQ water (Fig. 1B), indicating no particle aggregation in the co-crystal solution, and thus confirming a different aggregation state compared to the mixture and KLS alone that accounts for a substantial change to the physicochemical properties.

Such effects are especially relevant in models like ours, where cellular responses depend on transient exposure dynamics, not just steady-state solution composition. Thus, the “mechanisms” mentioned include release coordination, local bioavailability, and modulation of epithelial pathways, which together explain why the cocrystal behaves differently from a simple solution of its components.

3. The authors should clearly highlight the drawbacks of 2DE like limited detection of low-abundance or hydrophobic proteins, and difficulty in resolving post-translational modifications, possibly leading to incomplete proteome characterization.

Response: We thank the Reviewer 1 and we agree. We have now clarified this in the revised manuscript. Please check lines 380-384 in the “Discussion” section for the new information.

4. Can the authors highlight potential next steps to confirm the role of oxidative stress pathways? ROS measurements? Antioxidant Enzyme Activity?

Response: We appreciate the Reviewer’s comment and we added the potential next steps in the ms. As reported in the ms (lines 318-324), in a previous study (https://doi.org/10.1016/j.biopha.2023.114845) we reported the biomolecular characterization of the chosen model, in which regarding oxidative stress the following markers have been assessed: 4-HNE; CATALASE; SOD1. On this basis, potential next steps can include direct measurements of intracellular reactive oxygen species (ROS) as well as the activity and expression levels of antioxidant enzyme glutathione peroxidase (GPx), using both enzyme activity assays and Western blotting. These experiments will provide functional evidence to support or refine the proteomic findings, clarifying whether the observed protein modulations reflect true oxidative imbalance or compensatory responses. Moreover, by integrating quantitative ROS data with redox-sensitive signaling markers, such as Nrf2 or HO-1, it is possible to dissect the dynamics between oxidative damage and cellular defense mechanisms in our model system. This comprehensive approach will be essential to establish causality rather than correlation.

5. The Ethanol-Induced Injury Model has many limitations they do not fully replicate human gastric ulceration complexity, which includes factors like H. pylori infection and chronic NSAID use. Can the authors replicate this in-vitro and study its effects through a proteomic analysis.

Response: We thank the Reviewer 1 for the suggestion and we agree that the ethanol-induced injury model only partially reflects the multifactorial nature of human gastric ulceration complexity. In future studies we will improve the complexity of gastric ulceration using other approaches to compare the data.

Reviewer 2

1. Figure 1- Please include molecular weight for marker in the figure.

2. Figure 3.2- Please include molecular weight for marker in the figure.

3. In depth in vitro studies will be necessary to establish the model and to check its effect. Can the authors include figures of cell line model in supplement figure to show the effect of co-crystal

Response: We would like to thank Reviewer 2 for the time spent reading our manuscript and for the comments provided that helped in improving our article. We tried to address all the points raised.

Comments

1. Figure 1- Please include molecular weight for marker in the figure.

Response: We thank the Reviewer 2 for the comment. We modified Figure 1 providing the molecular weights in the figure. Please see Figure 1.

2. Figure 3.2- Please include molecular weight for marker in the figure.

Response: We would like to thank the Reviewer 2 for the suggestion. We specified molecular weights in Figure 3.2 as requested. Please check Figure 3.2.

3. In depth in vitro studies will be necessary to establish the model and to check its effect. Can the authors include figures of cell line model in supplement figure to show the effect of co-crystal.

Response: We thank the Reviewer for the comment. We now provided supplementary figures describing the effects of the co-crystal in the chosen model as suggested. Please see Figure S2 (S4_file).

Journal Requirements

When submitting your revision, we need you to address these additional requirements.

Response: We would like to thank the Editorial Office. We tried to address all the points raised.

Comments

1. Please ensure that your manuscript meets PLOS ONE's style requirements, including those for file naming. The PLOS ONE style templates can be found at https://journals.plos.org/plosone/s/file?id=wjVg/PLOSOne_formatting_sample_main_body.pdf

and https://journals.plos.org/plosone/s/file?id=ba62/PLOSOne_formatting_sample_title_authors_affiliations.pdf

Response: We thoroughly revised the manuscript according to PLOS ONE’s guidelines.

2. If any supporting files for review show as item type ‘other’ please change to item type ‘supporting info’ as the reviewer does not have access to these ’other’ files

Response: All the supporting files were originally loaded as item type “supporting information”.

3. Thank you for stating the following in the Competing Interests section: [Laura Brandolini, Andrea Aramini and Marcello Allegretti are employees of Dompe pharmaceutical Spa].

We note that you received funding from a commercial source: [Dompe pharmaceutical Spa]

Please provide an amended Competing Interests Statement that explicitly states this commercial funder, along with any other relevant declarations relating to employment, consultancy, patents, products in development, marketed products, etc.

Within this Competing Interests Statement, please confirm that this does not alter your adherence to all PLOS ONE policies on sharing data and materials by including the following statement: ""This does not alter our adherence to PLOS ONE policies on sharing data and materials.” (as detailed online in our guide for authors http://journals.plos.org/plosone/s/competing-interests). If there are restrictions on sharing of data and/or materials, please state these. Please note that we cannot proceed with consideration of your article until this information has been declared.

Please include your amended Competing Interests Statement within your cover letter. We will change the online submission form on your behalf.

Response: We provided the “Competing interests” as required. However, we filled the “Funding” section with the “Not applicable” statement as we received no fund at all for this project.

4. When completing the data availability statement of the submission form, you indicated that you will make your data available on acceptance. We strongly recommend all authors decide on a data sharing plan before acceptance, as the process can be lengthy and hold up publication timelines. Please note that, though access restrictions are acceptable now, your entire data will need to be made freely accessible if your manuscript is accepted for publication. This policy applies to all data except where public deposition would breach compliance with the protocol approved by your research ethics board. If you are unable to adhere to our open data policy, please kindly revise your statement to explain your reasoning and we will seek the editor's input on an exemption. Please be assured that, once you have provided your new statement, the assessment of your exemption will not hold up the peer review process.

Response: We would like to thank the Editorial Office for the suggestion. Indeed, we confirm that the data have already been loaded on Zenodo to avoid time loss (DOI: https://doi.org/10.5281/zenodo.14979855) and the dataset will be made “Not-restricted” as the paper publishing is confirmed. Moreover, we now provided the DOI of the dataset in the “Statistical Analysis” and “Availability of data and materials” sections. Please check lines 190 and 407, respectively.

5. PLOS requires an ORCID iD for the corresponding author in Editorial Manager on papers submitted after December 6th, 2016. Please ensure that you have an ORCID iD and that it is validated in Editorial Manager. To do this, go to ‘Update my Information’ (in the upper left-hand corner of the main menu), and click on the Fetch/Validate link next to the ORCID field. This will take you to the ORCID site and allow you to create a new iD or authenticate a pre-existing iD in Editorial Manager.

Response: We would like to thank the Editorial Office for the suggestion. We provided ORCID iDs in the Editorial Manager.

6. PLOS ONE now requires that authors provide the original uncropped and unadjusted images underlying all blot or gel results reported in a submission’s figures or Supporting Information files. This policy and the journal’s other requirements for blot/gel reporting and figure preparation are described in detail at https://journals.plos.org/plosone/s/figures#loc-blot-and-gel-reporting-requirements and https://journals.plos.org/plosone/s/figures#loc-preparing-figures-from-image-files. When you submit your revised manuscript, please ensure that your figures adhere fully to these guidelines and provide the original underlying images for all blot or gel data reported in your submission. See the following link for instructions on providing the original image data: https://journals.plos.org/plosone/s/figures#loc-original-images-for-blots-and-gels.

Response: We would like to thank the Editorial Office for guiding us in this process. We provided all the original uncropped and unadjusted images underlying all blot and gel results in the submission files, item “Other”.

Once again, we would like to thank the Reviewers and the editors for their time and we hope that now our research article is suitable for publication in Plos One journal.

Your sincerely,

Prof. Annamaria Cimini

Dr. Marcello Allegretti

Attachment Submitted filename: Response to Reviewers.docx

# Peer review: Decision Letter 1

**DOI:** 10.1371/journal.pone.0328496.r003

**Authors:** Anisha DSouza

Evaluation of the gastrotolerability of ketoprofen, lysine, and gabapentin co-crystal administration in an in vitro model of gastric epithelium: a proteomic update

PONE-D-25-12165R1

Dear Dr. Cimini,

We’re pleased to inform you that your manuscript has been judged scientifically suitable for publication and will be formally accepted for publication once it meets all outstanding technical requirements.

Within one week, you’ll receive an e-mail detailing the required amendments. When these have been addressed, you’ll receive a formal acceptance letter and your manuscript will be scheduled for publication.

An invoice will be generated when your article is formally accepted. Please note, if your institution has a publishing partnership with PLOS and your article meets the relevant criteria, all or part of your publication costs will be covered. Please make sure your user information is up-to-date by logging into Editorial Manager at Editorial Manager®  and clicking the ‘Update My Information' link at the top of the page. If you have any questions relating to publication charges, please contact our Author Billing department directly at authorbilling@plos.org.

If your institution or institutions have a press office, please notify them about your upcoming paper to help maximize its impact. If they’ll be preparing press materials, please inform our press team as soon as possible -- no later than 48 hours after receiving the formal acceptance. Your manuscript will remain under strict press embargo until 2 pm Eastern Time on the date of publication. For more information, please contact onepress@plos.org.

Kind regards,

Anisha DSouza, Ph.D (Tech)

Academic Editor

PLOS ONE

Additional Editor Comments (optional):

Thank you, authors, for addressing the reviewers comments. We have reviewed the responses, and it appears that the reviewers are satisfied with them.

Reviewers' comments:

Reviewer's Responses to Questions

**Comments to the Author**

Reviewer #1: All comments have been addressed

Reviewer #2: All comments have been addressed

**********

2. Is the manuscript technically sound, and do the data support the conclusions?

Reviewer #1: Yes

Reviewer #2: Yes

**********

3. Has the statistical analysis been performed appropriately and rigorously?

Reviewer #1: N/A

Reviewer #2: Yes

**********

4. Have the authors made all data underlying the findings in their manuscript fully available?

The PLOS Data policy

Reviewer #1: Yes

Reviewer #2: Yes

**********

5. Is the manuscript presented in an intelligible fashion and written in standard English?

Reviewer #1: Yes

Reviewer #2: Yes

**********

Reviewer #1: (No Response)

Reviewer #2: (No Response)

**********

what does this mean? ). If published, this will include your full peer review and any attached files.

If you choose “no”, your identity will remain anonymous but your review may still be made public.

**Do you want your identity to be public for this peer review?** For information about this choice, including consent withdrawal, please see our Privacy Policy

Reviewer #1: No

Reviewer #2: No

**********

# Editor report: Acceptance letter

**DOI:** 10.1371/journal.pone.0328496.r004

**Authors:** Anisha DSouza

PONE-D-25-12165R1

PLOS ONE

Dear Dr. Cimini,

I'm pleased to inform you that your manuscript has been deemed suitable for publication in PLOS ONE. Congratulations! Your manuscript is now being handed over to our production team.

At this stage, our production department will prepare your paper for publication. This includes ensuring the following:

* All references, tables, and figures are properly cited

* All relevant supporting information is included in the manuscript submission,

* There are no issues that prevent the paper from being properly typeset

You will receive further instructions from the production team, including instructions on how to review your proof when it is ready. Please keep in mind that we are working through a large volume of accepted articles, so please give us a few days to review your paper and let you know the next and final steps.

Lastly, if your institution or institutions have a press office, please let them know about your upcoming paper now to help maximize its impact. If they'll be preparing press materials, please inform our press team within the next 48 hours. Your manuscript will remain under strict press embargo until 2 pm Eastern Time on the date of publication. For more information, please contact onepress@plos.org.

If we can help with anything else, please email us at customercare@plos.org.

Thank you for submitting your work to PLOS ONE and supporting open access.

Kind regards,

PLOS ONE Editorial Office Staff

on behalf of

Dr. Anisha DSouza

Academic Editor

PLOS ONE
